# A blockchain based deep learning framework for a smart learning environment

**DOI:** 10.1038/s41598-025-03688-z

**Published:** 2025-06-04

**Authors:** Shimaa Ouf, Soha Ahmed, Yehia Helmy

**Affiliations:** Information Systems Department, Faculty of Commerce and Business Administration, Helwan University, Cairo, Egypt

**Keywords:** E-learning, Smart learning environment, Blockchain, Smart contract, Ethereum, Deep learning, Interplanetary file system, Computer science, Information technology

## Abstract

In the contemporary digital age, education is no longer limited to traditional educational environments. Many educational institutions shifted to depend on the smart learning process but expressed concern about this solution due to its various challenges in securing the learning process and learners’ data. By virtue of the most recent technologies like blockchain and artificial intelligence, which played a significant role in solving many challenges that faced the educational sector and overcoming issues like fake certificates, manipulation, tracking learners’ activities, and predicting learners’ academic performance. The study proposed a smart framework based on blockchain and deep learning to enhance smart learning processes and provide solutions for challenges in the field. The framework is intended to store the learner’s data on the blockchain through the interplanetary file system and reap the benefits of securing the learner’s data and ensuring its integrity, as well as ensuring the confidentiality and authentication of the users through the wallets that are created on the Ethereum private blockchain platform. Then apply the deep learning model to this secured data to predict the learner’s performance. The smart contract functions also play a role in enabling the university to issue learners’ certificates that are stored on the blockchain to be available and verifiable by all the nodes in the network. Based on the experimental results, deep neural networks were used to model the encrypted data that was stored on the blockchain and predict the learner’s performance and achieved a high degree of accuracy (91.29%) and low loss (about 0.18) in comparison to other studies that depended on the centralized nature of the data. As well, the university blockchain’s functionality was tested, and it successfully returned all the functional requirements and showed its legitimacy.

## Introduction

E-learning has undergone a significant revolution since it has been considered essential in dire situations like the COVID-19 epidemic. The significance of this case was brought to light and significantly altered. Well-known e-learning systems have been employed in many nations. These online learning environments were given permission by UNESCO as a quick measure. Due to the issues with e-learning systems and how they influence various elements of the learning process, it also did not suggest these platforms as a long-term fix. The study discussed the challenges with e-learning that came up as well as viable solutions. While some studies used artificial intelligence and deep learning techniques to focus on e-learning challenges that related directly to the learner’s performance and evaluating the learner’s academic progress, other studies used blockchain technology and smart contracts as a solution to overcome other challenges related to fake certificates, result manipulation, and tracking the learning activities of the learners. Both the blockchain, artificial intelligence and deep learning technologies proved their ability to provide great capabilities in any sector, as well it is found that there are few studies that discuss the integration of blockchain and artificial intelligence in the e-learning, and there are some studies that showed the importance of integrating the blockchain and deep learning in many sectors other than the educational sector, From this point, the study proposed a smart framework based on the blockchain and deep learning to enhance the e-learning processes and provide solutions for the challenges in this field to turn it into a secured smart e-learning system. This integration could ensure data security, transparency and provide an automated and trusted smart learning environment.

The study intended to store the learner’s data on the blockchain through the interplanetary file system and reap the benefits of securing the learner’s data and ensuring its integrity, as well as ensuring the confidentiality and authentication of the users through the wallets that are created on the Ethereum private blockchain platform. Then apply the deep learning model to this secured data to predict the learner’s performance. The smart contract functions play a role in enabling the university to issue learners’ certificates that are stored on the blockchain to be available and verifiable by all the nodes in the network. Although focusing on applying the smart contract to the learning activities increases the automation of the learning process and ensures security and transparency between the learners and the professor in tasks such as assignment submission, with the blockchain and deep learning technology, the guests (employers) can trust the data recorded in the chain, as all the recorded data is immutable and the learner’s performance prediction is highly accurate. Based on the experimental results, artificial neural networks were used to model the encrypted data that was stored on the blockchain and predict the learner’s performance with a high degree of accuracy, in comparison to the other studies that used the same dataset but based on a centralized nature, as well as a low loss of the model, indicating the validity of the model because the recorded data is immutable and the learner’s performance prediction is highly accurate. The university blockchain’s functionality was then evaluated, and it successfully returned all the functional requirements and showed its legitimacy.

Blockchain technology offers permanent data storage since transactions can only be added to and cannot be changed or deleted^1^. Blockchain is a distributed peer-to-peer communication where anti-members cannot be proven to interact with each other without the need for a trusted authority; blockchain is also seen as a set of interrelated mechanisms that can provide unique features for system facilities^2^.

In 2017 Drescher emphasized that blockchains are effectively viewed as a digital storage system that is unrelated to the records stored in each block and recognized the following as the expressive features like it is immutable, time stamped, secure and transparent^3^. Ethereum is a blockchain platform that implements peer-to-peer transactions to achieve a conditional, automated transfer of assets and information, including not just financial information but also other types of information. In terms of market capitalization, Ethereum comes in second place after Bitcoin. Like Bitcoin 3, it makes use of a public blockchain and a similar consensus algorithm. Ether is the name of Ethereum’s cryptocurrency (ETH)^4^. Ethereum provides a decentralized ecosystem for developers to provide and develop applications using Solidity as a programming language and the Ethereum virtual machine (EVM)^5^. EVM is the main software program that deploys smart contracts and determines the status of the Ethereum network when each new transaction is added to the blockchain network^6^. Where smart contracts are considered encrypted boxes that store value and are only applied in specific states. Through the smart contract, you may also create transactions based on a specified condition, which is more powerful than what the Bitcoin application provides. Due to its added power, value awareness, and blockchain awareness^6^.

Smart contract is a software program that adds a new layer of information to transactions that are executed on the blockchain. The term “smart contract” was coined in 1994 by the American scientist Nick Szabo, who recognized that distributed ledgers, could be used for smart contracts. A smart contract is a programmable contract that can be automatically applied when predefined conditions are met. A smart contract is an agreement between parties involved in a transaction that holds each party accountable^7^.

Smart contracts that are implemented on the blockchain have approved contract terms that are translated into executable computer programs and ensure the correct execution of the contract. Logical connections between contract terms are also maintained in the form of logical flows within the program like the “if–else” statements, the execution of each contract is stored on the blockchain as an unalterable transaction^8^.

Large files cannot be effectively stored on blockchains. So, it is important to decide which data should be put on the chain and which should be kept off the chain. There are many off-chain data storage solutions designed.

To be friendly with the blockchain, such as Storj^9^, File Coin^10^, and IPFS^11^. These solutions share the idea of a peer-to-peer distributed file system, which encrypts and shreds data before distributing it to other network nodes for security and availability. One major problem with these systems is the lack of access control. These characteristics are crucial for the deployment of blockchain-based applications with off-chain data storage methods in operational and sensitive areas like the public and financial sectors^1^.

One drawback that drives the study towards these solutions is that as more data is distributed across the blockchain network, the blockchain swells. However, since the blockchain is distributed over multiple nodes, a lot of storage space is needed without having the functionality right away, especially if the node operator does not need to read every file maintained on the blockchain^1^. A study concludes that huge files should not be shared or stored on blockchains. Furthermore, apps may be supported while preserving the blockchain’s small size by employing file-sharing networks. Users can effectively distribute large files while still getting blockchain benefits. A file’s content can be securely identified using cryptographic hashes, which can be sent to the recipient to demonstrate who had access to the file and when. The interplanetary file system (IPFS), which combines file sharing with hashes, is an especially promising file-sharing platform for this purpose^12^.

After illustrating the blockchain, IPFS, and the smart contract, the study focuses on the second significant technology, which is deep learning.

A form of artificial intelligence system called an “artificial neural network” (ANN) is based on the composition and operation of biological nerve systems, or animal brains, and it may be applied to sophisticated supervised, unsupervised, or reinforcement learning algorithms. Artificial neural networks are still primitive in comparison to animal brains, despite learning to do some wonderful things (such as identify the faces of individuals). They usually involve only a few neurons, as opposed to the billions of neurons found in the human brain.

Artificial neural networks include three layers: an input layer that accepts environmental input in the form of various data sources, like pixels from photographs; at least one hidden intermediate layer, but sometimes much more; an output layer that presents the outcome. During the machine learning process, weights assigned to interconnections are changed in a reinforced learning process, enabling the artificial network of neurons to calculate outcomes for new stimulation^13^.

The network is referred to as a “deep neural network” when there are multiple hidden layers (DNN). A multilayer perceptron (MLP) learns by doing the following:

1. Forward propagation, where the input data (the training dataset) is propagated forward through the network to produce the output.
2. Errors are calculated by comparing network output to the desired output.
3. Backpropagation of errors, calculation of derivatives for each network weight, and model update.

We perform multiple iterations (epochs) of the earlier steps until we get sufficient resolution (reduce the error below the desired outcome). DNNs with seven to fifty layers are now often employed. The complexity of a network is influenced by the quantity of neurons, connections, and weights. Every weight denotes a variable that must be learned. The amount of weight used influences how difficult the training is^14^.

In 2023, An Interactive Dashboard for Education Abroad with Geospatial Information and Predictive Analysis, this paper concentrated on the creation of a comprehensive dashboard to aid students in identifying appropriate universities for their higher education abroad. The research underscored the obstacles that students encounter when seeking master’s programs, such as the selection of universities, the admission requirements, and other critical factors that influence their decisions. Investigates the use of augmented reality (AR) to improve hybrid (online and offline) educational systems. It tackles conventional education difficulties, such as the difficulty of teaching particular ideas in offline classrooms, by introducing an AR environment in which students may interact with 3D objects and sounds linked to their studies. This method seeks to make learning more interesting and dynamic, allowing students to acquire knowledge more intuitively and effectively^15^.

In 2023, a survey on AR-based digitization for smart education systems this study examined the incorporation of augmented reality (AR) into digital education to improve the learning experience. It emphasized that online education necessitates a substantial amount of effort from both students and instructors, which can be psychologically taxing. The proposed AR-based system is designed to simplify intricate educational concepts by providing a 3D learning environment that is interactive. Engaging with digital objects and audio that are pertinent to their subjects can enhance the effectiveness and immersion of the learning experience for students. This hybrid educational approach, which integrates both online and offline components, facilitates the comprehension and retention of information^16^.

The study concludes that activity-based learning is a highly effective student-centered approach that improves educational outcomes. ChatGPT can be a valuable tool for enhancing learning experiences through interactive and personal assistance. However, its implementation requires careful consideration to maintain educational integrity and foster critical thinking skills^17^.

All these researchers have examined the role of modern technology within the educational system, and we will also explore the potential impact of deep learning on the e-learning system.

Deep learning models were used to predict the student’s performance. A dropout prediction model for Massive Open Online Courses (MOOCs) was introduced in 2017 with the goal of predicting whether students would finish the course. Due to the problem of a high MOOC failure average. They said that the conventional methods relied on manually extracting the characteristics, which are costly, time-consuming, and impractical for incorporating fresh datasets from various platforms or modules. Therefore, this study proposed a deep neural network model that depends on various measures, such as the number of students enrolled in a particular course, the number of different events, and the number of days spent taking the course, and that is an integration of convolutional neural networks and recurrent neural networks that can automatically generate features from raw data^18^.

In 2017, An analysis of how deep learning techniques may be used to forecast students’ performance and a comparison of the study to other machine learning methods that have long been in use Based on the consistency of the student’s performance and his prior terms, they used feed-forward neural networks as well as recurrent neural networks to produce a model to accurately predict the student’s grade point average (GPA). They found that since recurrent neural networks had memory and took student performance consistency into account, they performed more accurately than feed-forward neural networks^19^.

In 2019, for MOOC academics and providers, predicting dropouts—or figuring out if a student is leaving a course, is a major worry. As a result, the study’s definition of the dropout prediction issue is calculating the amount of material a student can learn across the course’s entire curriculum. A dropout rate prediction model makes use of a recursive neural network. Because it only has a few parameters and therefore requires less processing, the recursive neural network won the comparison between it and long-term short-term memory. Statistics show that the model’s prediction accuracy is much higher than that of a normal machine learning model, demonstrating that it performs better on a variety of tasks. Additionally, two contributions to the dropout prediction were made by the suggested model. First off, rather than real learning activities, the dropout forecast is based on curricular success. Second, a generic dropout prediction model based directly on the resource access log may be obtained by employing the recursive neural performance methods with a learning resource representation layer instead of the weekly activity feature. Consequently, in the initial weeks, the suggested model produced an accurate forecast^20^.

In 2021, research was conducted with the purpose of improving early learner performance prediction to reduce the student’s chances of failing. To solve their imbalanced, limited dataset, they employed both synthetic minority over-sampling procedures and a dense deep learning model. The study demonstrated that deep learning is a useful method for predicting the student’s performance in only two courses, mathematics, and Portuguese, with high accuracy based on the provided small dataset, which contained the student’s demographic characteristics, family situation, way of life, and educational information^21^.

In 2021, a model for predicting dropout was established based on the student’s performance from educational data, and the study effectively forecasted the student’s risk of failing a course by employing bidirectional long- and short-term memory networks^22^.

The following Table 1 summarizes all the studies about using deep learning to predict students’ performance.

**Table 1:** Deep learning for student’s performance prediction.

References	Research goals	Dataset size	Algorithm	Layers	Accuracy
Patil et al.^20^	Predict the performance of a student in prior years Predict student’s GPA	Small	Bidirectional recurrent neural network	4 hidden	76%
		2000 records		2 outputs	
Wang et al.^19^	Design a dropout prediction model in a MOOC	Large dataset	Convolutional neural network (ConRec)	1 hidden	87.42%
		120,542 records		1 output	
Sun et al.^21^	Develop a dropout prediction model for MOOCs based on course content and resource access logs	Large dataset	GRU-RNN	1 output	Not mentioned
		12,847 records		1 output	
Aslam et al.^22^	Predict the student’s final academic achievement for two courses only according to their daily activity logs	Small-imbalanced	Dense DL model	6 hidden	0.96 for the mathematics course 0.93 for the Portuguese course
		395 records for mathematics course 649 records for Portuguese course		1 output	

## The role of the blockchain and smart contract in the e-learning system

Smart educational systems and blockchain work better together. As a result, smart education offers the potential for accuracy, transparency, security, and effectiveness in the educational process^23^. According to a study, using the transparency and non-tampering characteristics of the blockchain to incorporate a collaborative education industry scheme using the Hyperledger framework would serve as an innovative and essential solution for universities and businesses to work collaboratively and share information^24^. The obstacles to e-learning listed among the numerous studies conducted in various nations are summarized in Table 2 below, which highlights some of the concerns that must be resolved to enhance the entire e-learning process.

**Table 2:** Blockchain and e-learning challenges.

References	Security	Student prediction system	Student records and scores	Intellectual property rights	Student privacy	Course sharing and credibility	Credential verification	Certificates	Evaluation security	E-learning assessment	User Interactivity	System interoperability	Online examination	Copyrights issues	Test result data storage	Results tampering	User authentication	Identity misuse	Huge students’ population
^25^	×	×	√	√	√	√	√	√	×	×	×	×	×	×	×	×	×	×	×
^23^	√	×	×	×	×	×	×	×	√	×	×	×	×	×	×	×	×	×	×
^26^	×	×	×	×	×	×	×	√	×	√	×	×	×	×	×	×	×	×	×
^27^	×	×	×	×	×	×	×	√	×	×	×	×	×	×	×	×	×	×	×
^28^	√	×	×	×	×	×	×	×	×	×	√	√	×	×	×	×	×	×	×
^29^	×	√	×	×	√	×	×	√	×	×	×	×	×	√	×	×	×	×	×
^30^	×	×	√	×	×	×	×	×	×	×	×	×	√	×	×	×	×	×	×
^31^	×	×	√	×	×	×	×	√	×	×	×	×	×	×	×	×	×	×	×
^32^	×	×	×	√	×	×	×	×	×	×	×	×	×	×	×	×	×	×	×
^33^	×	×	√	√	×	×	√	√	×	×	×	×	×	×	×	×	×	×	×
^34^	×	×	×	×	×	×	×	×	×	×	×	×	×	×	×	×	√	√	×
^35^	√	×	×	×	×	×	×	√	×	×	×	×	×	×	×	×	√	×	×
^36^	×	×	×	×	×	×	×	×	×	×	×	√	×	×	×	×	×	×	×
^37^	×	×	√	×	×	×	×	√	×	√	×	×	×	×	×	√	×	×	√

## The convergence of blockchain and the deep learning techniques

Because of the difficulties that deep learning techniques face due to the centralized nature of data storage that lacks traceability and transparency, resulting in an increase in a vulnerable single point of failure as well as issues with data integrity that may corrupt the training model, the convergence of deep learning and blockchain has received a lot of attention. With its decentralized efficacy and shared ledger technology, blockchain technology is used as a solution to tackle these problems^38^.

According to Shafay et al., the integration of deep learning and blockchain can have many benefits, such as automated and reliable decision-making, effective data market administration, data security, improved model building for prediction, model sharing, and increased resilience of deep learning-based systems. A powerful, long-lasting, and distributed infrastructure for the crucial data that deep learning applications would acquire, examine, and employ might be made possible by combining deep learning and blockchain. Increased system resiliency, automated decision-making, accurate forecasting, and effective data market management, as well as improved data security, automatic decision-making, and progressive evaluation^38^.

Based on four factors, including blockchain type, consensus algorithm, deep learning model, and data, there are various applications for blockchain-based deep-learning frameworks. There are applications in control of traffic, the internet of vehicles, and healthcare.

Healthcare: To predict disease transmission rates and offer relevant preventive measures, deep learning models may be trained using healthcare data. The patient profiles and disease data in the healthcare system helps the model predict the patients’ medical status. One point of failure might emerge from the central data storage system for patient profiles and diseases. Data protection from accidental or purposeful loss is ensured by blockchain^39^.

Traffic management: One of the most critical issues in cities is traffic, which may result in considerable losses in terms of money and the environment due to rising carbon dioxide emissions. One of the most popular techniques for determining traffic intensity using real-time data is crowdsourcing. However, due to concerns over human safety and centralized data storage, current crowdfunding solutions are ineffective^40^.

Internet of vehicles: The networking capabilities of contemporary devices have been improved by the introduction of the Internet of Things as a vehicle instrumentation technology. Modern transportation networks frequently equip vehicles with devices, sensors, and virtual agents that enable data sharing and communication. By integrating blockchain into the Internet of Vehicles network, data may be transmitted between entities in a secure manner^41^.

As mentioned above, the convergence of blockchain technology and deep learning was presented in many areas rather than e-learning.

## Research problem

The study presented artificial intelligence and deep learning approaches in e-learning and served as a literature review for the study. Additionally, it presented the significance of blockchain and smart contracts in the e-learning system, and it showed the lack of studies that have consolidated artificial intelligence and blockchain in some learning process components, as well as the absence of studies on applying deep learning with blockchain in the e-learning fields. Because of the importance of e-learning and the recent trend and reliance on it. The study focused on providing a smart framework based on blockchain and deep learning for enhancing the e-learning system and providing a smart learning environment.

## Research methodology

Due to the importance of blockchain and deep learning, this study introduces a proposed framework that is composed of three phases for integrating the blockchain and deep learning and illustrating the transformation of the learning system into a smart, transparent, and secure e-learning system.

**Fig. 1:**
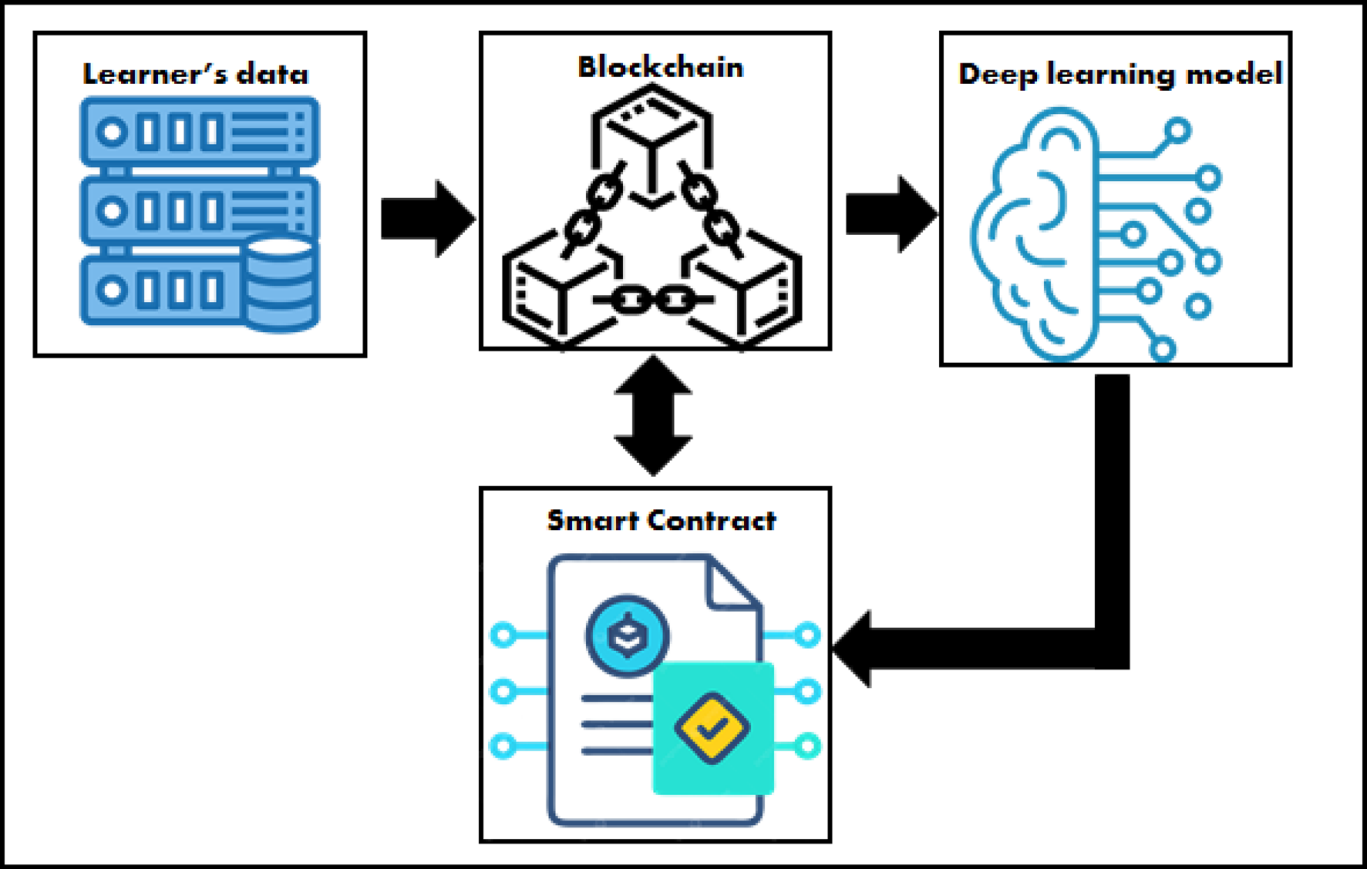
The proposed framework.

Figure 1 depicts the framework’s main phases in a general view. The first phase is to create blockchain architecture and store the learners’ data to ensure data security and decentralization.

The second phase is implementing deep learning techniques for predicting the learner’s academic performance. The third phase involves using smart contracts to perform transactions between nodes on the blockchain.

## The university’s blockchain nodes

**Fig. 2:**
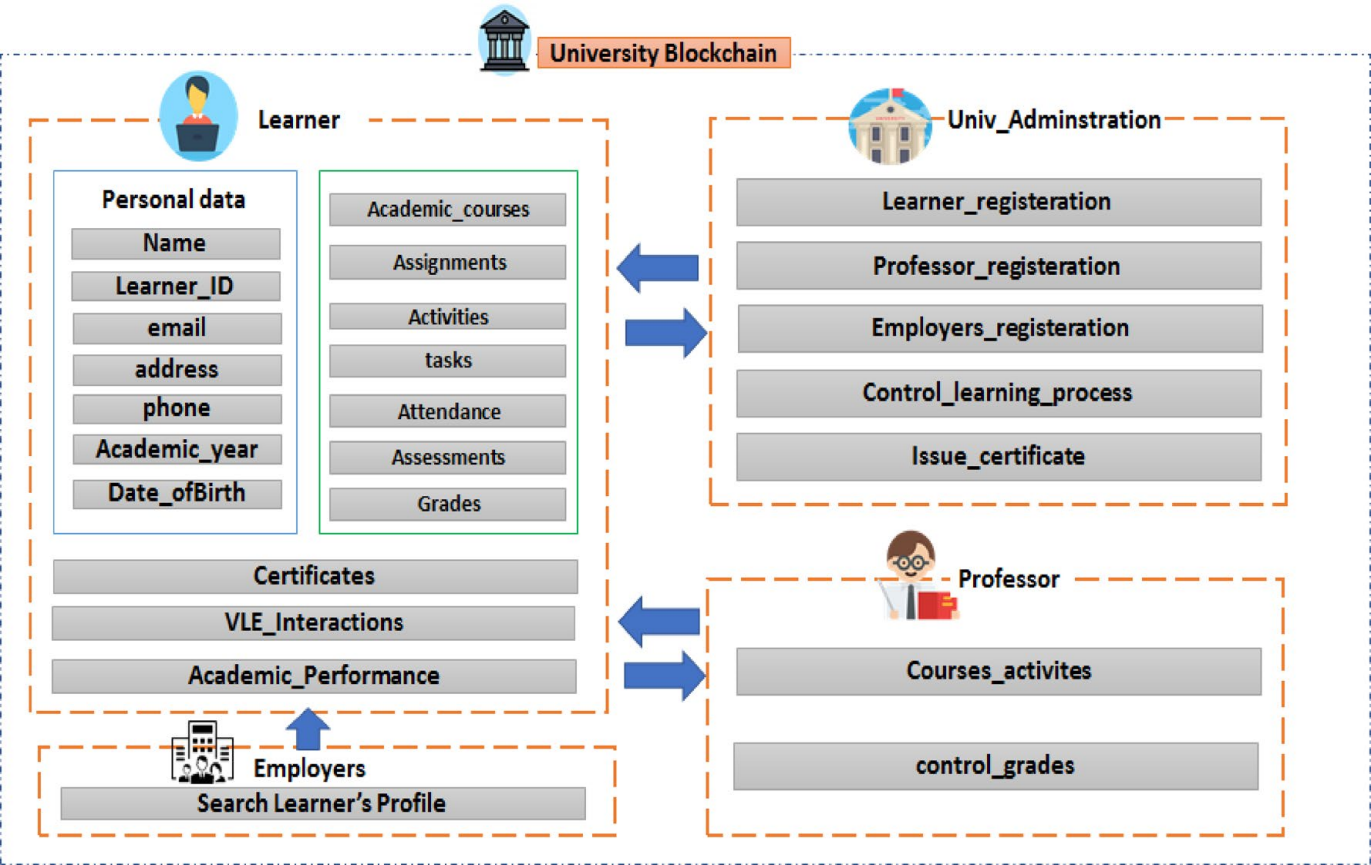
The University’s blockchain components.

The development of the university blockchain includes the learning process components as mentioned in Fig. 2 where the learner’s data is sent to the deep learning model to use the deep neural networks to predict the learner performance and then to store the learner’s status in the blockchain to ensure its reliability.

Figure 2 illustrates the university’s blockchain components, which include the main participating nodes like the university administration, learners, professors, and employers.

The learner’s data is stored in the blockchain, like personal information, course activities, submissions, virtual learning interaction, and academic learning performance status. The university administration is responsible for learner registration, professor registrations, and any employers accessing the university network, controlling the learning process activities, and issuing certificates for the learners. The professor is responsible for the courses’ activities, such as tasks, assignments, control grades, or any assessments that evaluate the learner’s performance during the learning process. Finally, employers or any organization can have permission from the university administration to access the network and search or view only the learner’s profile to get the most qualified or best-matched candidate for a job. The node types concerning the roles in the university blockchain can be categorized into:

1. University Administration (Univ_Admin).
  - Read/write learner registration data.
  - Read/write professor registration data.
  - Read/write university rules to govern blockchain network access.
  - Set up a new node to the network.
  - Issue learner’s certificate
  - Add block.
2. Learner node.
  - Read/write course registration.
  - Read/write some personal data.
  - Read only certificate/learner transcript.
3. Professor (faculty member) node.
  - Read/write courses list data.
  - Read/write tasks for learners.
  - Read/write some personal data.
  - Read only learner’s certificate/learner’s transcript.
  - Add block.
4. Employer (guest) node.
  - Read only certificate/learner’s transcript.
  - Read only courses list and curriculum.

## Nodes registration on the blockchain network

Each node should register on the blockchain network to deal with the others and make transactions on the network. Briefly each node will get access to the network through its address, the blockchain wallet that includes private and public keys. Algorithm 1 in Table 3 shows a sample for the node’s registration in the blockchain network of the learner and professor.

**Table 3:** Algorithm 1, nodes registration.

**Algorithm 1**	**Nodes_Registeration**
**Input**	**Learner_academicID or NationalID, Professor registration request**
**Output**	**A newly registered learner to access the blockchain network.** **A newly registered Professor to access the blockchain network**
1:	check ← check_identity ();
2:	IF identity == true
3:	Learner_ID ← generate learner_ID ();
4:	(${L}_{Pub}$,${L}_{Pri}$) ← generate Learner_Public_Private_Keys ();
5:	Blockchain_Address ← generate Learner_Blockchain_Address (${L}_{Pub}$,${L}_{Pri}$);
6:	Instructions ← send Instructions();
7:	Blockchain_wallet ← create_Blockchain_wallet (${L}_{Pub}$,${L}_{Pri}$);
8:	**else**
9:	Reject learner registeration;
10:	checkProfessor_ID ← generate Professor_ID ();
11:	(${P}_{Pub}$,${P}_{Pri}$) ← generate Professor_Public_Private_Keys ();
12:	Blockchain_Address ← generate Professor_Blockchain_Address (${P}_{Pub}$,${P}_{Pri}$);
13:	Instructions ← send Instructions();
14:	Blockchain_wallet ← create_Blockchain_wallet (${P}_{Pub}$,${P}_{Pri}$);

The following sections illustrate the three phases of the proposed framework in more detail.

### Phase 1: store learner’s data on the blockchain

**Fig. 3:**
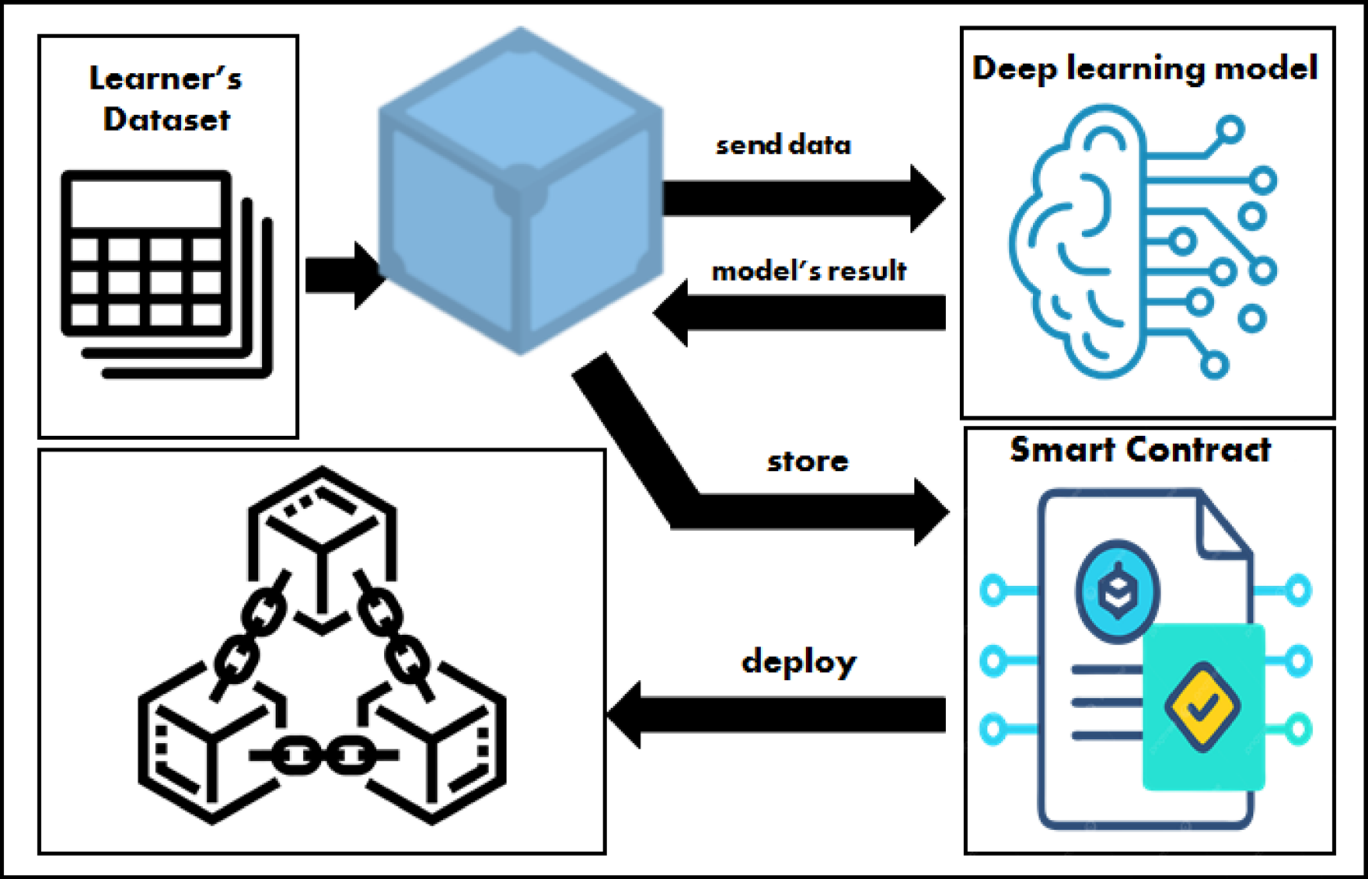
The integration of blockchain and deep learning in the smart learning environment.

Figure 3 shows the integration of the blockchain and deep learning techniques in providing a smart learning environment.

The figure illustrates in more detail the sequence of storing the learner’s data into the blockchain through the IPFS, where the IPFS generates a hash for the data and enables it to be distributed, decentralized, secured, and immutable. Then the deep learning model works on this data to predict the learner’s academic performance. The smart contract plays a significant role in the framework for making transactions between nodes and storing them in the blockchain network to ensure the security of the system. The smart contract is written in Solidity through the Solidity remix IDE, and the blockchain development architecture of the university blockchain will be linked as a workspace in the Ganache platform, which acts as an Ethereum private blockchain and is integrated with the MyEtherWallet platform to enable the nodes’ interactions through their wallets. All these processes will be discussed later in the implementation section.

#### The learner’s dataset in the blockchain

The learner’s data and all the learning activities and interactions are stored on the blockchain through the pnterplanetary file system (IPFS) protocol, an attempt to link all computing devices with the same file system made by the peer-to-peer distributed file system known as IPFS. The IPFS creates a hash for every file and delivers a high-performance content-addressed shared file system with content-addressed hyperlinks. IPFS offers responses to the issue of file storage in the blockchain since it can efficiently store large data in a distributed manner^42^. At its essence, IPFS is a timestamped file system that can store files and keep track of their changes over time. IPFS creates a distributed file system based on the hashing function that enables the file to be hidden from outside parties by defining how a file flows throughout the network.

#### Learner’s dataset acquisition

The dataset was acquired from a well-known publicly available dataset named “Open University Learning Analytics”^43^ It includes a 32,593 record about students’ demographics and their virtual learning interactions by means of their clickstream to show their interaction and behavior. The following Tables 4 and 5 describe information about the acquired dataset.

**Table 4:** Features of the acquired dataset.

Number of learner’s records	32,593
Number of courses	22
Student’s clickstream	10,655,280
Measurement	Student behavior

**Table 5:** Dataset’s attributes description.

Characteristics	Attributes	Description
Student_information	code_module	The code that identifies the registered module
	code_presentation	The code of the registered module presentation that was submitted during specific time
	id_student	Unique student identifier number
	region	The place where the student lives in it
	highest_education	The student’s academic level
	imd_band	The place where the student makes the presentation from it
	age_band	The range of the students ages
	num_of_prev_attempts	How many times did the student enrolled in the module course
	studied_credit	The total number of credit hours for the modules that the student is currently studying
	disability	Whether the student is disable or not
	final_result	The final grade for the module presentation
Courses	code_module	The code that identifies the registered module
	code_presentation	The code of the registered module presentation that was submitted during specific time
	module_presentation_length	The time taken for completing the presentation
Student_assessment	id_assessment	Unique assessment identifier number
	id_student	Unique student identifier number
	date_submitted	The assessment submission date
	is_banked	The status of the assessment result transferred from the previous presentation
	score	The student’s grades in the assessment, ranged from 0 to 100
Student_registeration	code_module	The code that identifies the registered module
	code_presentation	The code of the registered module presentation that was submitted during specific time
	id_student	Unique student identifier number
	date_registeration	The date on which the student enrolled on the course
	date_unregisteration	The date on which the student withdraws from the course
VLE	id_site	The unique identifier number of the module
	code_module	The code that identifies the registered module
	code_presentation	The code of the registered module presentation that was submitted during specific time
	activity_type	The function accomplished with the module
	week_from	The start-date for the virtual interaction
	week_to	The end-date for the virtual interaction
Student_vle	id_student	Unique student identifier number
	code_module	The code that identifies the registered module
	code_presentation	The code of the registered module presentation that was submitted during specific time
	id_site	The unique identifier number of the module
	date	Date of the student interaction and click streaming
	activity_type	The function accomplished with the module
	sum_click	The total number of clicks that the student made

The dataset includes tables such as student information, courses, student registration, student assessment, vle, and student_vle. The tables are linked together by the relationship between them; where the student is linked with his or her demographic information and the registration of the courses, the dataset includes the results of the students’ assessments and logs of the virtual learning interaction. Table 5 shows the attributes in each table and their descriptions. In this study, all the data were merged into one table and named “learner performance”. taking into consideration the relationships between the tables.

### Phase 2: use deep learning to predict the learner’s performance

**Fig. 4:**
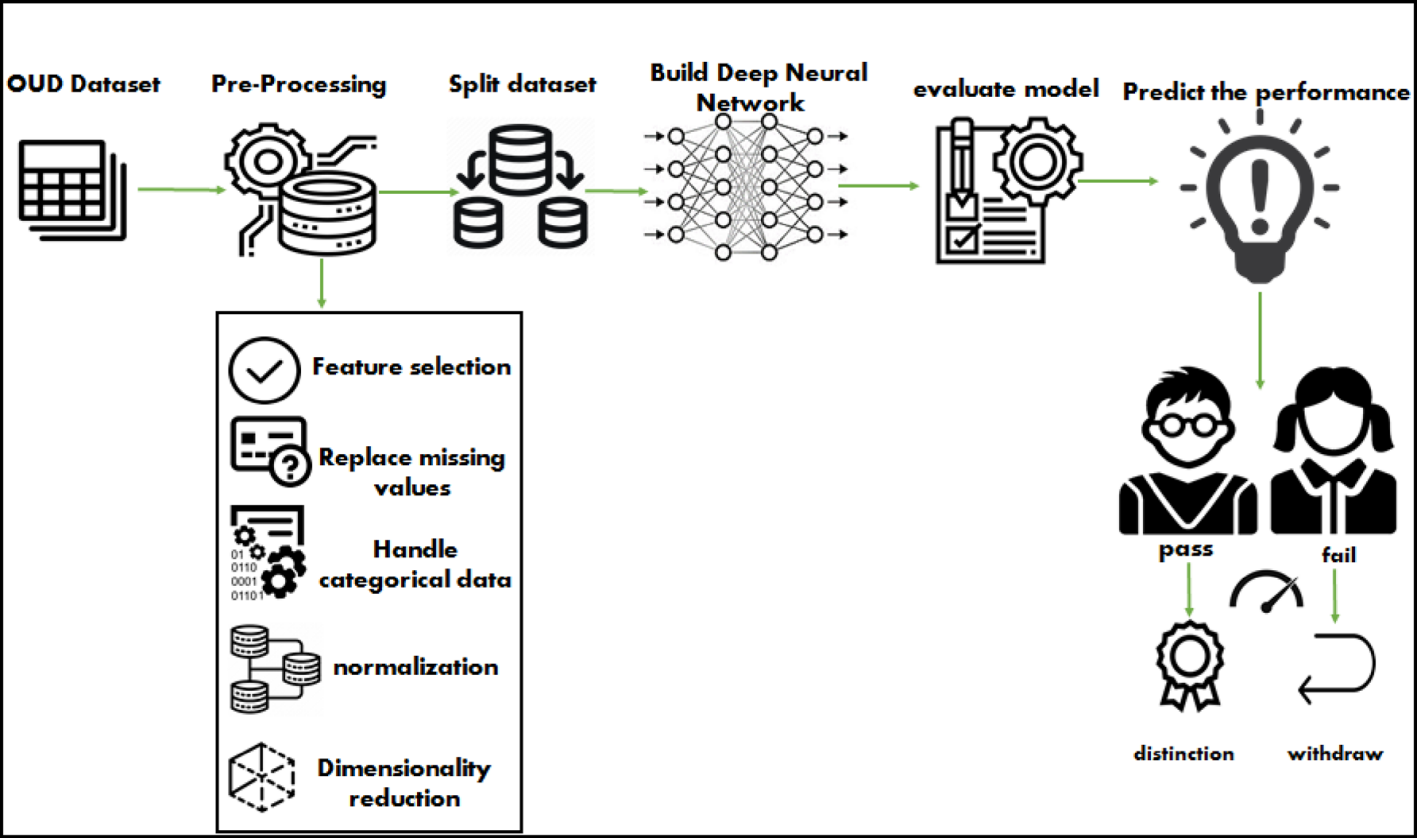
The proposed deep learning framework.

This phase will focus on the suggested framework for forecasting a learner’s performance in a virtual learning environment using deep learning techniques. Figure 4 depicts the four primary layers that are used to achieve the result of the performance prediction.

layer1: Dataset acquisition.

layer2: pre-processing.

layer3: split dataset.

layer4: build the deep neural network (learner’s performance prediction).

Layer 1, known as dataset acquisition, contains the data discussed in detail in the preceding section. Layer 2 is the pre-processing stage; this is regarded as the most crucial phase before beginning any method since it pertains to all the raw data transformation and preparation before it is input into the deep learning model^44^. This study’s preprocessing technique includes five steps: feature selection, missing value replacement, categorical data handling, normalization or feature scaling, and dimensionality reduction.

*Step 1* Feature selection.

Feature selection is a technique for restricting your model’s input variables by picking the relevant data and eliminating noise. It is the process of picking acceptable features for your deep learning model based on the type of issue you are seeking to solve^45^.

*Step 2* Replace missing values.

There are several causes of missing values, including completely random missing data and partially unpredictable missing data. These might all result from a system failure during data collection or a human error during data pre-processing. Before evaluating the data, missing values must be addressed since failing to do so might result in a biased or incorrect conclusion. There are several methods for handling missing values. Such as the constant value, the mean, the median, and the mode. The mean is used to provide an average value and is recommended when the data distribution is biased. The median can only be used with numerical data, whereas the mode may be used with both numerical and categorical data to get the most frequent value^46^. In this study the constant value is used and the mode as it gets the most common values.

To calculate the mode.

Equation 1: The mode’s equation^46^

1 $${\text{Mode}} = {\text{l}} + \left[ {\frac{{F_{m} - F_{1} }}{{2F_{m} - F_{1} - F_{2} }}} \right] \times h$$

The symbols represent, L: the lower limit of the class’s model; H: size of the class interval (assume all the classes to be equal); Fm: frequency of the class’s model; F1: Frequency of the class preceding the modal class; F2: Frequency of the class succeeding the modal class.

*Step 3* Handle categorical data.

Since neural networks work with matrices of real numbers, we must discover a technique to convert categorical data into matrices of actual numbers before we can use neural networks to analyze qualitative data. A determined method for expressing categorical values using matrices of real numbers must be used as a starting point. It entails getting qualitative data ready for neural network application. Before entering qualitative variables into a neural network, we must first translate them into numerical values using an algorithm. These techniques are sometimes referred to as “distributed approximations”, “interpretations”, or “encodings”^47^.

*Step 4* Normalization.

The dataset’s data was normalized to enhance the effectiveness of deep learning techniques for activity detection. From its techniques, the MinMaxScaler, which is used to translate and scale each feature independently by the specified range on the training set. The effects of deep neural network (DNN) normalization methods on the categorization of data activities^48^.

*Step 5* Dimensionality reduction.

This step entails making use of a generic problem’s highly correlated properties to significantly decrease the problem’s dimensionality and, thus, its computing complexity without introducing significant error^49^.

After that, Layer 3 is about splitting the dataset to ensure the quality and accuracy of the model by using the hold-out method. Layer 4, Building the deep neural network. The deep neural network model is employed to learn from the input features, which represent the learning activities of the learners, and predict who will pass and indicate that there is a distinction or failure and indicate that it is preferred to withdraw to not affect academic grades. The deep neural network is an artificial neural network that is from the family of deep learning algorithms that include multiple hidden layers between the input layer and the output layer^13^.

### Phase 3: employ the smart contract

The study focused on employing smart contracts to show the secured interaction between the nodes, which seeks to provide a smart learning environment. As well as adding transactions to the blockchain network. The smart contract will help the university administration store learner’s data in the blockchain network and allow the authorized participants to view these academic data. It will also show the interaction between the learner and the professor in receiving and submitting the course activities, then the interaction between the university administration and the learner in issuing the certificate and receiving it. Finally, it shows the transactions between the employer (the guest) and the learners to view their secured and transparent academic achievements.

#### Transactions between the professor and the learner

**Fig. 5:**
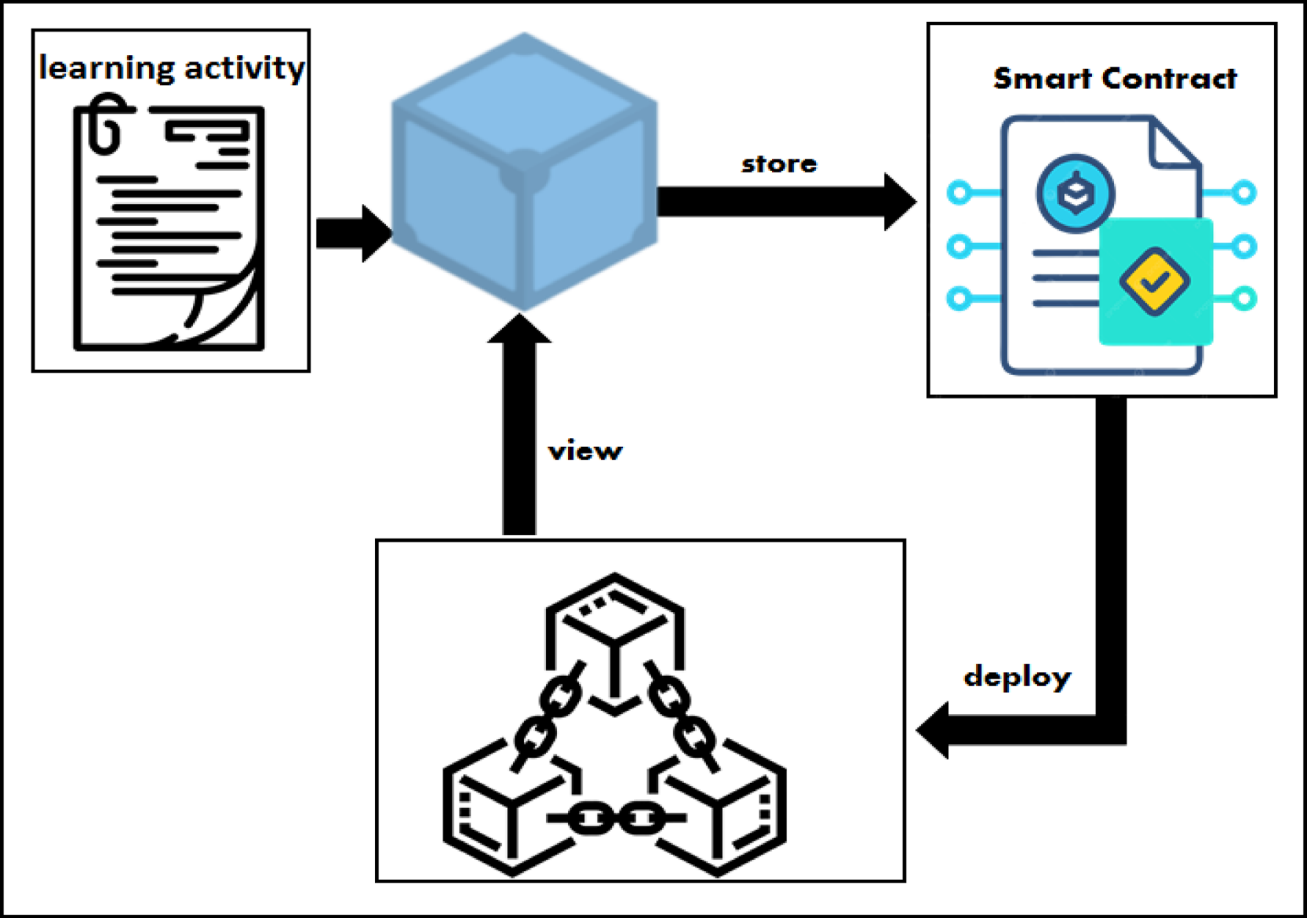
How the learning activity uploaded in the blockchain.

Figure 5 shows how the professor uploads a learning activity, such as an assignment or any task for the learner, on IPFS, how the hash of the file is used in the smart contract to be sent to the learner, how the contract is deployed on the network on the MyEtherWallet platform, and how the transaction is added to the blockchain network and viewed on Ganache.

Algorithm 2 in Table 6 discusses the function of sending the assignment to the learner through the smart contract, it shows the interaction and flow of messages between the professor and the blockchain network, where the professor signs the transaction using his private key before adding it to the network and then sends the contract address to the learner. On the other hand, when the learner receives the contract address, he or she can deal with the contract and view the learning activity sent to him.

**Table 6:** Algorithm 2, send assignment to the learner.

Algorithm 2	Send Assignment or task to learner
**Input**	Assignment file
**Output**	Assignment files send to the learner
**1:**	Assignment_file ← Create_hash (Assignment_file);
**2:**	Blockchain_Wallet ← Assignment_file_data (ByteCode, GasLimit);
**3:**	Sign_Contract ← ${P}_{Pri}$ (sign_transaction);
**4:**	Request ← Deploy_contract;
**5:**	Successful_request ← Contract_Deployed;
**6:**	Transaction ← Add_transaction();
**7:**	Send_ContractAddress ← SendtoLearner(LearnerAddress);

Algorithm 3 in Table 7 discusses the function of receiving the assignment activity by the learner through the smart contract.

**Table 7:** Algorithm 3, receive assignment from the learner.

Algorithm	**Learner Interacts with Assignment/task Contract**
**Input**	Learner’s Address, Assignment/task Contract Address
**Output**	Assignment file
**1:**	Validate ← check_Assignment_Contract_Address_Validity;
**2:**	If Assignment Contract Address == true;
**3:**	Request ← Successful_contract_Interaction;
**4:**	else
**5:**	Request ← Failed_Contract_Access;
**6:**	select_function ← Return(ViewAssignment);

#### The university issues learner’s certificate

**Fig. 6:**
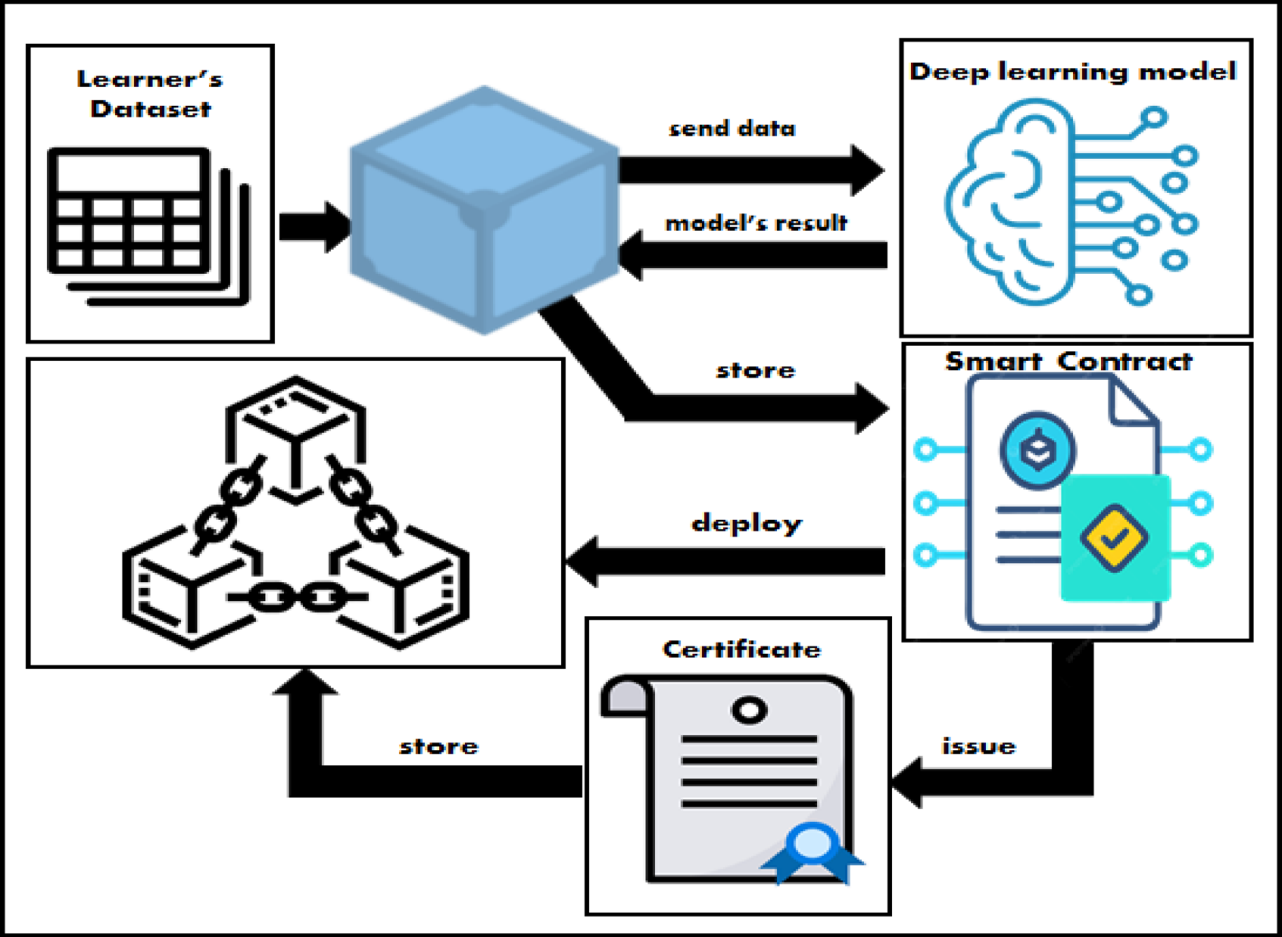
University issue learner’s certificate.

The university seeks to issue the learner’s certificate securely. Figure 6 depicts the steps taken by the university administration (univ_admin) to store the learner’s dataset in the distributed network IPFS. The stored encrypted data is modelled by deep learning techniques to predict the learner’s academic performance in accordance with the learner’s academic data. The learner’s data is stored on the blockchain by the smart contract. Also, it issues the learner’s certificate based on the model’s result, and the transaction is signed using the univ_admin private key. The transaction is then stored on the block and added to the blockchain using ganache.

The process of issuing the learner’s academic certificate is going to be between the university node and the learner through the smart contract functions deployed in the blockchain network.

Algorithm 4 in Table 8 shows the interactions between the university administration, and the learners in the network and illustrates the process of adding certificate issuance transaction to the blockchain network.

**Table 8:** Algorithm 4, learner’s certificate issuance.

Algorithm 4	Issue Certificate
**Input**	Learner’s data, Certificate data
**Output**	Add a course certificate for the learner
**1:**	Certificate ← SmartContract (CertificateData);
**2:**	Blockchain_Wallet ← CertificateData (ByteCode, GasLimit);
**3:**	Sign_Contract ← $\overline{{UA }_{Pri}}$ (sign_transaction);
**4:**	Request ← Deploy_contract;
**5:**	Successful_request ← Contract_Deployed;
**6:**	Transaction ← Add_transaction();
**7:**	Send_ContractAddress ← SendtoLearner(LearnerAddress);

#### The learner interacts with the certificate contract

Algorithm 5 in Table 9 illustrates how the learner interacts with the certificate contract address through the network and receives the returned result.

**Table 9:** Algorithm 5, learner interact with the certificate.

Algorithm 5	**Learner Interacts with Certificate Contract**
**Input**	Learner’s Address, Certificate Contract Address
**Output**	Learner’s certificate details
**1:**	Validate ← check_Certificate_Contract_Address_Validity;
**2:**	If Certificate Contract Address == true;
**3:**	Request ← Successful_contract_Interaction;
**4:**	Else
**5:**	Request ← Failed_Contract_Access;
**6:**	select_function ← Return(Certificate);
**7:**	Transaction ← Add_transaction();

It shows the interaction between the learner node and the blockchain network, the learner can access the network through the learner’s address and enter the certificate contract address. The system checks if the address is invalid, so it returns a failed access response but if the address is valid, it allows the learner to access the contract and select the contract functions.

#### Guest node interacts with the learner’s data

The employer, which acts as a guest node, interacts with the university network, and can view the learner’s profile and certificate upon the univ_admin’s approval.

Algorithm 6 in Table 10 presents how the guest, or the employer node, gets access to the blockchain network to view the learner’s overall data, performance status, or academic certificate.

**Table 10:** Algorithm 6, guest interaction in the network.

Algorithm 6	**Employer (Guest) views learner’s certificate**
**Input**	Employer’s Address, Certificate Contract Address
**Output**	Learner’s certificate details
**1:**	Request ← access_network;
**2:**	Approve_request ← UA(Approve_access_request);
**3:**	Request ← view_learner_certificate;
**4:**	Enter_Certificate_Contract_Address;
**5:**	Validate ← check_Certificate_Contract_Address_Validity;
**6:**	If Certificate Contract Address == true;
**7:**	Request ← Successful_contract_Interaction;
**8:**	else
**9:**	Request ← Failed_Contract_Access;
**10:**	select_function ← Return(Certificate);

It shows that the guest or employer node first requests to join the network, and the university administration should approve the request. Upon this approval, a blockchain address is generated, as well as a blockchain wallet that includes the private and public key. When a guest or an employer obtains the learner’s certificate contract address and enters it into the network, the system verifies that the contract address is valid so that the employer can access the learner’s certificate as well as the learner’s data for the learning activities. But if the address is invalid, it shows failed access to the contract.

## Implementation

This section discusses the actual development of the blockchain network, and the implementation of the proposed smart framework based on blockchain and deep learning, which is divided into two main parts. The first part is about the general blockchain network development, and the second part shows the integration of the blockchain with deep learning techniques for a smart learning system as well as, smart contract deployment.

Implementing the proposed framework is divided into two main parts: Part 1, which is the general blockchain development. It includes two phases. Phase 1 related to how to build the blockchain architecture, while phase 2 related to how to make the blockchain function. Then, Part 2, for implementing the proposed framework, is comprised of three phases, which are: storing learners’ data in the blockchain, apply the deep learning techniques and employ the smart contract.

### Part 1: general blockchain development

This section illustrates the general development of blockchain architecture, which is composed of two phases, Phase 1 is building the blockchain architecture, and phase 2 is about how to allow the blockchain network to perform the general working functions such as mine blocks, get the blockchain, check the blockchain validation, decentralize the blockchain, add transactions, and create a smart contract.

#### Phase 1: build the blockchain architecture

First, a general blockchain is created with its components, which should be found inside each block. The blockchain is decentralized on several computers or servers. To implement the blockchain, we used the Anaconda IDE, which is considered a studio that includes a bunch of tools to deal with. The blockchain was created using Python as a programming language and PyCharm. PyCharm is a scientific development environment for the python. Before the creation of the blockchain, we installed some important tools inside the Anaconda, such as:

- The flask web framework, which is used to build web applications containing the blockchain, will be used online on any server.
- The request package, to run some requests, such as mining some blocks or adding new transactions inside the blockchain.
- Postman, which is a user-friendly HTTP client, is used to interact with the blockchain and make some requests.

To start building the blockchain, important libraries should be imported first, like the DateTime library, because each block has its own timestamp, the exact date on which the block was created or mined. The essential hashlib library was used to hash the blocks, and it works with the hash function. From the JSON library, the dump’s function was used to encode the blocks before hashing them. Finally, the Flask library was used to enable the Flask class, which was used to create the web application’s objects. In addition, the jsonify function was used to return messages when we interacted with the blockchain via Postman, such as displaying the response to any requests to get the blockchain or mine a new block for the blockchain, and the jsonify function was used to return key information of the created block in a JSON format, such as the index of the block, the proof of the block, and the previous hash attached to the new block.

#### Phase 2: blockchain functions

Phase 2 of Part 1 of the proposed framework is applied to prove the implementation of the blockchain. Some functions were made to get the state of the blockchain and to mine new blocks in the blockchain through the Postman interface, which is a user-friendly interface.

##### Mine a new block

The first function inside the blockchain is to add a new block to the chain.

##### Decentralizing the blockchain

**Fig. 7:**
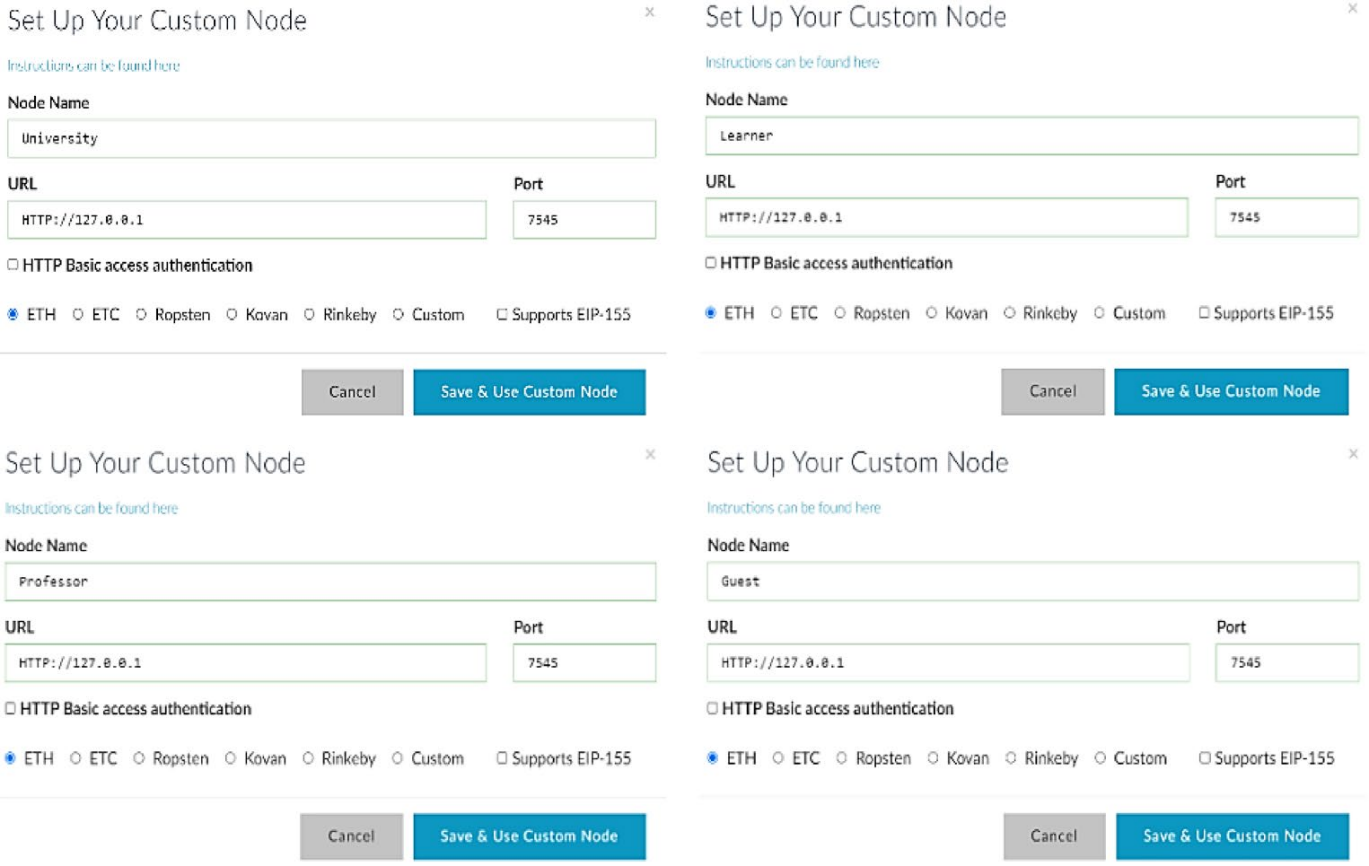
Setting up the functional nodes in the blockchain.

Decentralizing the blockchain network means setting up functional nodes in the Ethereum blockchain network through the MyEtherWallet (MEW) platform, where it acts as a client-side interface that is used for creating nodes’ accounts as wallets and shows the interaction between the nodes in the blockchain network. Figure 7 depicts a collection of functional nodes configured in the MyEtherWallet platform. It shows the setup of the university node in the network, the setup of the professor node in the network, the setup of the learner node in the network, and finally, the setup of the guest (employer) node in the network.

After creating the nodes and ensuring their connection to the decentralized network, each node from the various categories will access the myetherwallet to activate their wallet and obtain the keystore file, which contains the account address as well as the private and public keys. Each node should activate the wallet in the MEW by creating it and receiving the account address and the private and public keys.

##### Check validation

The blockchain functionality should be checked to see if it is valid or not before continuing in the next steps, such as configuring this architecture, linking it with the Ganache platform as the workspace of the private Ethereum platform, and viewing the blockchain from this interface.

##### Add transactions

This process considered how to add transactions to the block inside the blockchain. This process is applied through smart contracts. The process will be discussed in detail in phase 3 of Part 2, which will take up the process of employing the smart contract and how the transactions are added to the blockchain.

### Part 2: integrate the blockchain and deep learning for a smart learning system

The second part of the implementation is about integrating the blockchain with deep learning, as mentioned previously in the illustration of the framework. The phases that are needed to achieve the goal of integrating these two important technologies are:

- phase1: Store learner’s data in the blockchain.
- phase2: Implement the deep learning techniques for predicting the learner’s academic performance.
- phase3: Employ the smart contract.

#### Phase 1: store learner’s data in the blockchain

As mentioned before in the previous sections that illustrate the characteristics and features of the learner’s dataset, this phase proposes that the learner’s dataset will be stored on the blockchain using the interplanetary file system (IPFS) protocol, which provides solutions for the file storage problem, as it can store large files efficiently, which is a file-storage system suitable for the Internet’s next generation. IPFS is a document—oriented file system that can store files and keep track of their changes throughout time. IPFS creates a distributed file system that is inaccessible to outside parties by defining how a file passes over the network.

##### Storing learner’s dataset in the IPFS

**Fig. 8:**
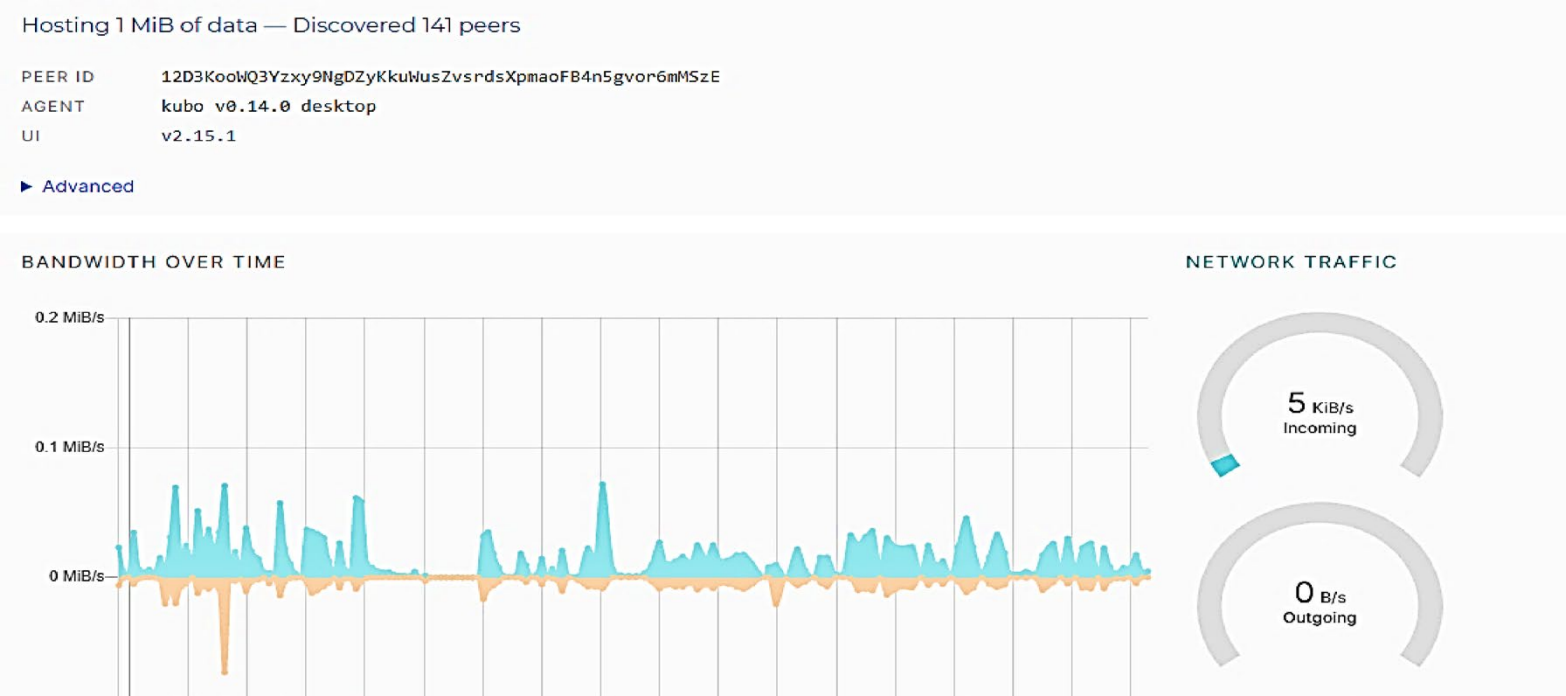
Peer nodes in the IPFS.

To protect the data, it is stored in an encrypted format on an IPFS server under the appropriate supervisory authority (University) where a hash of the data is generated. The following Fig. 8 shows the IPFS and the total number of connected peers at the time.

**Fig. 9:**
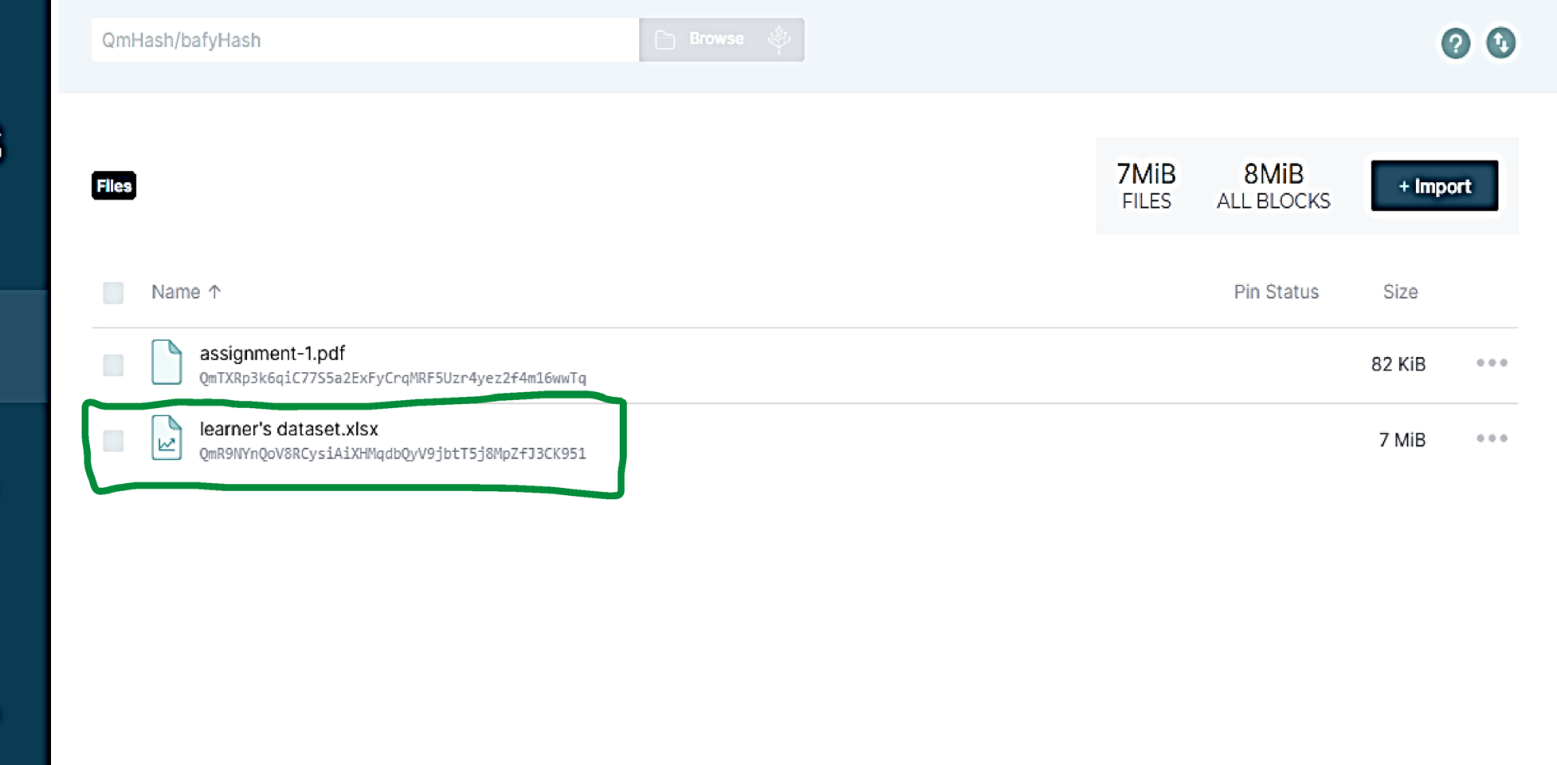
The uploaded learner’s dataset.

First, the learner’s dataset used in the deep learning model was stored on the IPFS and a hash was generated. The dataset includes about 32,592 records. The following Fig. 9 show the learner’s data storage and its hash in the IPFS.

In phase 2, the implementation is applied on the stored learner’s data from the blockchain.

#### Phase 2: implementing the deep learning techniques

This phase relates to applying the deep learning techniques for predicting the academic performance of the learners in accordance with the dataset stored on the blockchain, as mentioned in the previous section. The implementation of the deep learning techniques in this study depends on four layers.

##### Layer 1: dataset acquisition

This layer is illustrated in detail in the previous section.

##### Layer 2: pre-processing

This layer includes five steps: feature selection, replacing missing values, handling categorical data, normalization or feature scaling, and dimensionality reduction.

*Step 1* Feature selection.

**Fig. 10:**
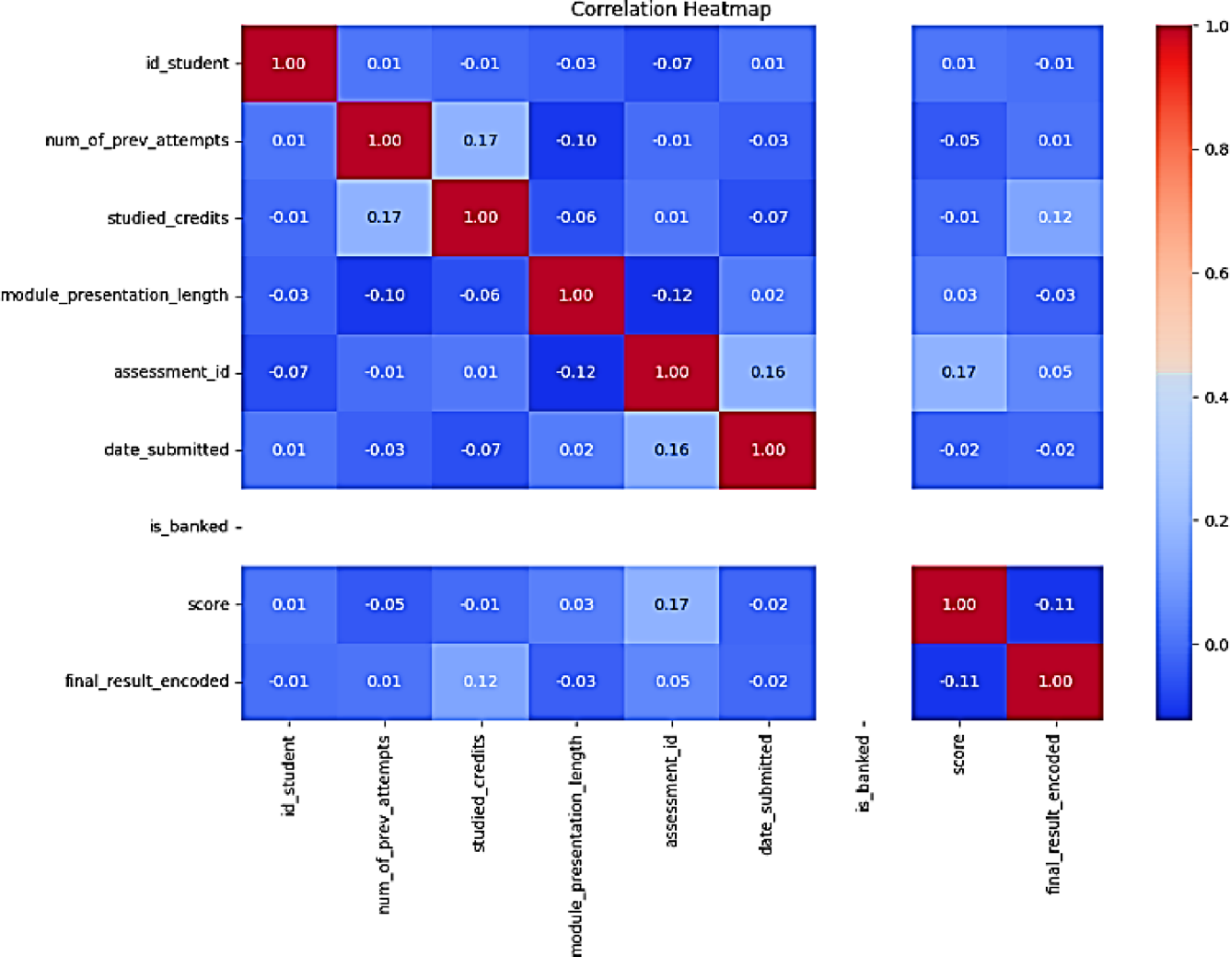
Heat map for feature’s selection.

In this process, we determine the most important features that will be utilized in the model as input and output features. We drop the unneeded features such as code_presentation, id_student, module_presentation_length, assessment_id, imd_band, and age_band from the input and consider the output feature to be the “final result”, by using the correlation coefficient for the numeric variable. The following Fig. 10 shows the heat map of the feature selection.

*Step 2* Replace missing values

The dataset has been cleaned, with some values addressed using the mode, as mentioned earlier, while the remaining null values have been replaced with [0].

*Step 3* Handle categorical data

**Fig. 11:**
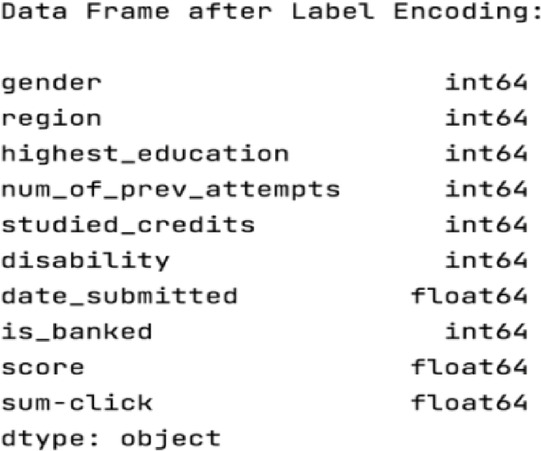
Handle categorical data.

In this step, all the text-value data is converted into numerical values, features like (gender, region, highest_education, and disability) are handled by the LabelEncoder. The following Fig. 11 shows the result of applying the label encoder algorithm for converting the categorical data into integer data.

*Step 4* Normalization

**Fig. 12:**
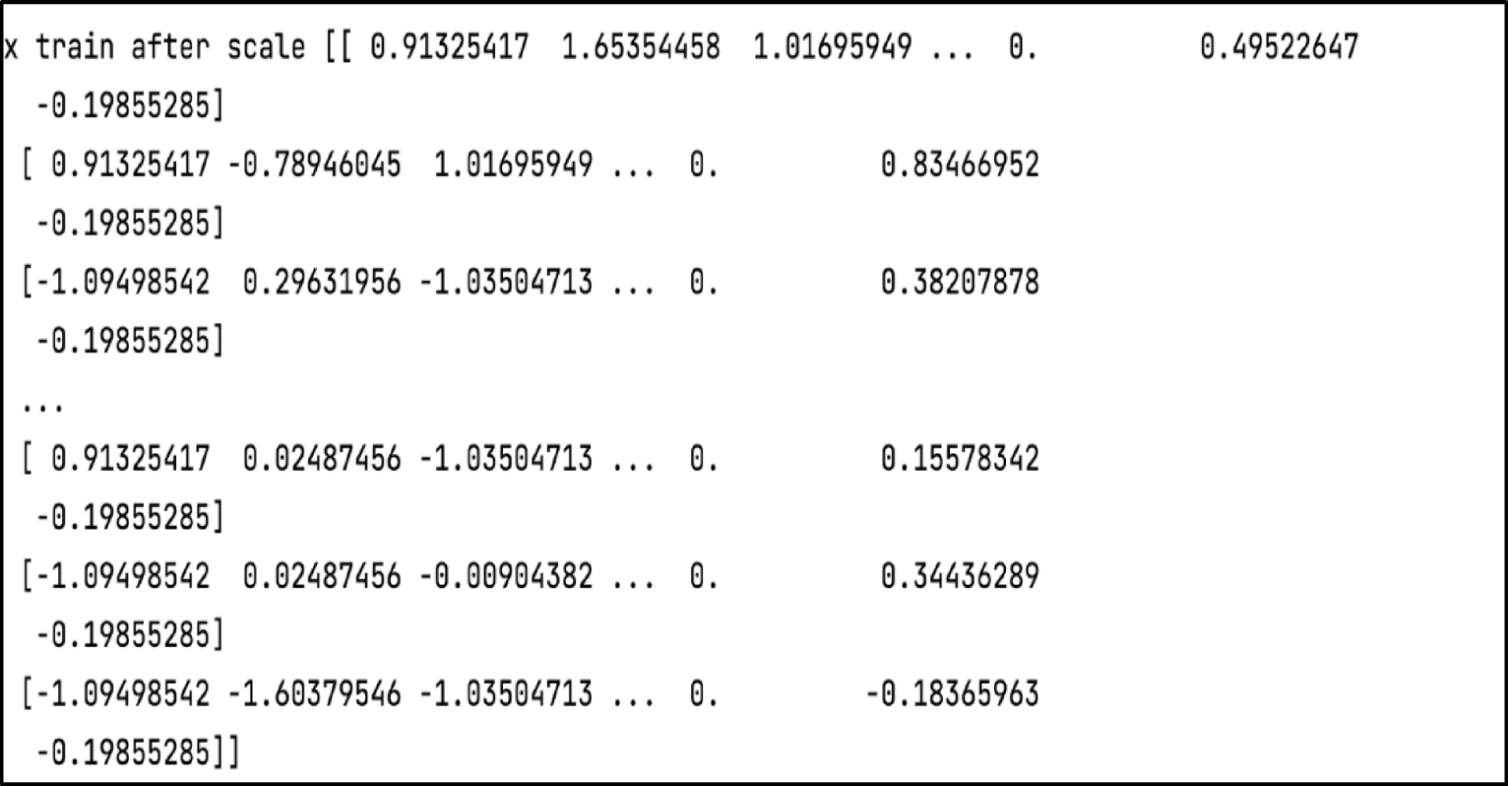
The training data after scaling.

**Fig. 13:**
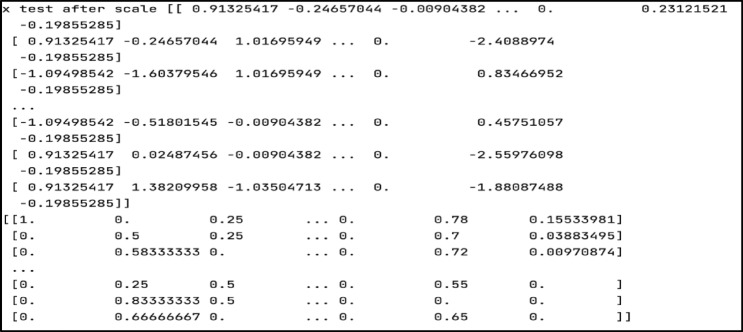
The testing data after scaling.

Normalization is a sort of feature scaling that involves scaling features in a range of 0 to 1 in order to make the model more consistent and allow it to predict outcomes reliably. In this study, the dataset is rescaled using the MinMaxScaler. Scaling is performed twice: after training and after testing the dataset. Figure 12 displays the train after the scale, whereas Fig. 13 shows the test after the scale.

*Step 5* Dimensionality reduction

Dimensionality reduction is the process of minimizing the dimensions of a huge dataset while retaining as much information as feasible. As a result, the predictive model’s quality improves while training time is reduced. The principal component analysis has four major steps: standardization, covariance, eigenvectors, and eigenvalues. Each of these steps is critical in the dimensionality reduction process^50^. In this work, the principal component analysis (PCA) is used to minimize the amount of features in the dataset while maintaining its quality to ensure model correctness.

##### Layer 3: split the dataset

The hold-out method is used to split the data into 90% train data and 10% test data and to increase the model’s accuracy.

##### Layer 4: build the deep neural network

**Fig. 14:**
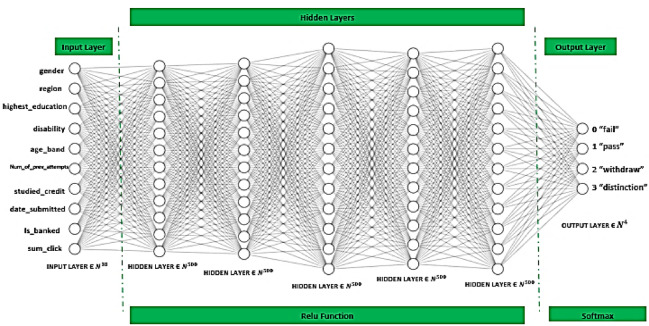
The proposed deep neural networks.

The proposed learner’s performance prediction framework was implemented using the Python programming language through the PyCharm professional IDE. Several libraries were used, such as Sklearn, Keras, and Matplot. The following Fig. 14 illustrates the deep neural network model for predicting the learner’s performance in the virtual learning environment. The model consists of 7 layers: the input layer, 5 hidden layers, and the output layer. The input layer includes 10 neurons, which refer to the target features in the model. The hidden layers retain five hidden layers with a total of 500 neurons in each hidden layer, which are activated with the rectified linear unit function (Relu). The output layer consists of 4 neurons, where the softmax function is utilized for the output layer, and the best loss function accompanied by it is the sparse categorical cross-entropy function. All of these were performed with the utilization of the Adam optimizer.

The 4 output neurons refer to the student performance prediction, where 0 means fail, 1 means pass, 2 means withdraw, and 4 means distinction.

#### Phase 3: employing the smart contract

This phase captures the algorithms used for implementing the proposed framework and the process of deploying the smart contract that plays a vital role in integrating deep learning into the blockchain by adding transactions to the blocks inside the blockchain.

The implementation of the smart contracts takes place by using the remix solidity IDE, myetherwallet platform, and ganache interface that shows the insertion of the transactions into the block inside the Ethereum blockchain network. There will be two ways to deal with the contract, either to deploy and initiate the contract or interact with an existing contract.

In the proposed study, after applying the deep neural networks and predicting the learner’s academic performance status as well, the model proved its efficiency in comparison to other studies, the data will be stored on IPFS and stored on the blockchain for use in the smart contract.

##### Store the dataset hash on the Ethereum using smart contract

To complete and finish the process of storing learner’s data in the blockchain, the smart contract is used to store the dataset hash on the Ethereum blockchain network and to enable any node to access or view the data. The contract is written in the Solidity language and interacts with the blockchain via the Solidity remix IDE and Myetherwallet store the IPFS dataset’s hash in the blockchain through the smart contract and compiling the contract.

##### Deploy the contract on the Ethereum blockchain

- The IPFS learner’s data and model’s result hash are stored in the learner’s profile in Myetherwallet on Ethereum blockchain platform. The byte code should be inserted, and the gas limit is determined automatically to deploy the contract and perform the transaction.
- The Ganache platform is used to obtain the keys of the node to connect with the private Ethereum blockchain; in this case, the learner’s private key is retrieved from the ganache to access the wallet at any time.
- Then the private key is used for the university’s node network to deploy the contract in myetherwallet.
- The transaction being signed and published in the network.

## Broadcast the transaction on the Ethereum network

When the contract is deployed, the transaction is published on the Ethereum network using the Ganache platform. The information about the account address, the number of transactions, and transaction timestamp will appear on the platform. Also, the transaction data, its hash, and the contract address that is connected to it inside the block in the chain will be available on the platform.

### Access the contract functions on the blockchain network

The user interacts with the contract’s function through the myetherwallet by adding the contract address, the application binary interface (ABI) of the contract.

### Issue the learner’s academic certificate

The remix Solidity IDE is used to create a smart contract enabling the university (univ_admin) to issue the academic certificate of the learners. The learner can interact with this and view the certificate from the university’s node network on the Ethereum blockchain. The following steps show the issuance of the certificate by the university through the smart contract.

### Create the certificate smart contract

The solidity programming language is used to create the certificate smart contract, then it is deployed, and the transaction is completed. Through specific steps.

1. The university node enters the byte code of the certificate contract, and the gas limit is identified automatically. It then chooses to access the wallet through the private key.
2. The contract is signed by the university private key, and then the contract is deployed.
3. The result of the deployed contract will be a new block mined with a transaction added to it in the blockchain network.

### Transactions between the professor and the learner

According to the proposed framework that was mentioned in the previous chapter, there are learning activities performed by the professor to evaluate the learner during the learning process in a secure manner. The following steps illustrate how these learning activities take place.

1. Upload the learning activity data into the blockchain.

The professor’s assignment that is provided to the learners is uploaded to IPFS.

2. Store the Assignment hash into blockchain

The smart code is used for storing the IPFS-Assignment hash into the blockchain through the smart contract.

3. Deploy the assignment contract.

The contact is deployed similarly to previous contracts by entering the contract’s byte code and gas limits.

4. The process of submitting the answers by the learners.

After viewing the assignment, learners should submit their responses using the same security and verification procedures as the assignment. The learner uploads the assignment to IPFS first, where a hash is generated for the file. After that, the learner uses the smart contract feature of the blockchain to upload the content identifier, or hash, of the answer’s file to the blockchain.

The learner interacts with the smart contract of the answer to add the hash of this submission as a transaction attached to the block on the network.

## Testing and evaluating the proposed model

This section intends to test and assess the functionality and advantages of utilizing the proposed framework of the smart e-learning system, which is based on blockchain and deep learning, to determine whether it meets the research objectives. It assures and shows the evaluation of blockchain security, followed by test scenarios that demonstrate the evaluation of functional components (smart contracts) and network node interactions. It also assesses the validity of transactions in the block inside the blockchain. Finally, it evaluates the deep neural network prediction model by assessing its accuracy and compares it to other research using the same dataset.

## Methodology of testing and evaluating the proposed framework

The phases of blockchain testing are composed of the initiation phase, the design phase, the testing phase, and the reporting phase. There’re phases of testing the blockchain^51^.

The testing phase is discussed here from the perspective of the study.

### Phase 1: initiation

In this step, we study and assess the functional requirements and business needs of blockchain architecture. This outlines the application’s behavior and how users will engage with the blockchain network. The following Table 11 summarizes the functional requirements of the blockchain and the participating nodes for each requirement.

**Table 11:** Functional requirements and business needs.

Functional requirements		Node
FR1	Create blockchain architecture	
FR2	Create nodes in the blockchain network	
FR3	Store learner’s data into the blockchain	Univ_admin
FR4	Predict the learner’s performance	
FR5	View learner’s data	Learner–professor–guest
FR6	Issues learner’s certificate	Univ_admin
FR7	View learner’s certificate	Learner–professor–guest
FR8	Create learning activities	Professor
FR9	Interact with learning activities	Learner

### Phase 2: design

The design phase includes the creation of test cases. During this phase, the test cases that follow the appropriate method are written to develop or extract test data that satisfies business needs from the preceding environment. The following Table 12 summarizes the test cases for assessing the blockchain, smart contract functionalities, and deep learning prediction model.

**Table 12:** Test cases for evaluation.

Test case no.	Test case type	Test case	Description	Expected result
TC1	Security	Verify the node’s account’s addresses are working	Create a new node address to access and interact with the contract	The node account address will be accepted and enables the node to interact with the contract
TC2	Smart contract test	How can the university ensure the validity of the stored learner’s data hash in the blockchain and share them?	Store learner’s data in the IPFS and create smart contract functions such as [get hash] to get the hash of the data and [send hash] to send the hash of the data to any node in the network	Hash of the learner’s data will be available
TC3	Smart contract test	How can the university issue a verifiable and non-tampered learner’s certificate?	The university node inserts the learner’s data as a transaction through the smart contract functions to issue the secure and non-tampered certificate	Insert learner’s certificate data
TC4	Smart contract test	How can the nodes view the learner’s certificate?	The learner or any node in the network can access the contract and enter the learner’s data to retrieve the certificate	View the learner’s certificate
TC5	Smart contract test	How can the learner interact with the learning activities on the blockchain?	The learner interacts with any learning activities uploaded by the professor through the smart contract functions	View assignment file
TC6	Smart contract test	How can the professor view the learner’s answers on the blockchain?	The professor interacts with the learner’s submission of any learning activities uploaded through the smart contract functions	View the assignment’s answers
TC7	Blockchain functionality	Check the visibility of the transactions inside the block in the network	Ensure the transaction validation through its visibility and mining in the blockchain network	Transaction found [mined] on the blockchain
TC8	Security	Check the integrity of the files in the blockchain network	Try to update the file and upload it again or try to delete the file from the decentralized network	The file cannot be deleted from all the nodes in the network once it is uploaded, and it cannot be uploaded again if it is updated
TC9	Model accuracy	Check the prediction model’s accuracy	Evaluate the deep learning prediction model’s accuracy	The Model’s accuracy [high accuracy] indicates a good model result
TC10	Model loss	Check the prediction model’s loss	Evaluate the deep learning prediction model’s loss	The Model’s loss [less loss result] indicates a good model result

### Phase 3: testing

In this phase, the test cases are implemented and tested, and the actual result is compared with the expected result.

#### Test case 1: verify the nodes account’s address

To verify the node’s existence in the network, the authentication of the users is activated and ensured when they can access their wallet at any time by using the Keystore downloaded file, or the private key.

The learner should enter the password to decrypt the wallet; once the message “The wallet is successfully decrypted” appears, the learner can access the wallet to determine the account address or keys.

Then the node can access the actual wallet which includes the account address, the keystore file, and the private key.

#### Test case 2: the university ensures the validity of the stored learner’s data hash in the blockchain and shares them

When the deployed contract is marked with the right mark and the hash appears, then it means that the smart contract can read the hash of the stored data, and it can be accessible. The input textbox of the send hash function, then approve the transaction from the Myetherwallet platform.

#### Test case 3: How can the university issue a verifiable and non-tampered learner’s certificate?

The university can access the certificate contract through its address and the application binary interface (ABI) to get access to the contract and deal with the function of issuing the learner’s certificate as a transaction on the blockchain network.

As mentioned in the illustrated sequence diagram in the previous sections, here are the steps for inserting the data into the blockchain for issuing the certificate.

The gas fees limit is automatically updated after the university node inserts data for issuing a certificate to the learner, and the transaction generation process.

Then the transaction will be successfully added to the blockchain.

Finally, all the blocks and transactions made by the university node will appear on the network.

#### Test case 4: How can the nodes view the learner’s certificate?

By selecting the certificate function, any node (for example, the learner or the guest) can enter personal information to view the academic certificate.

**Fig. 15:**
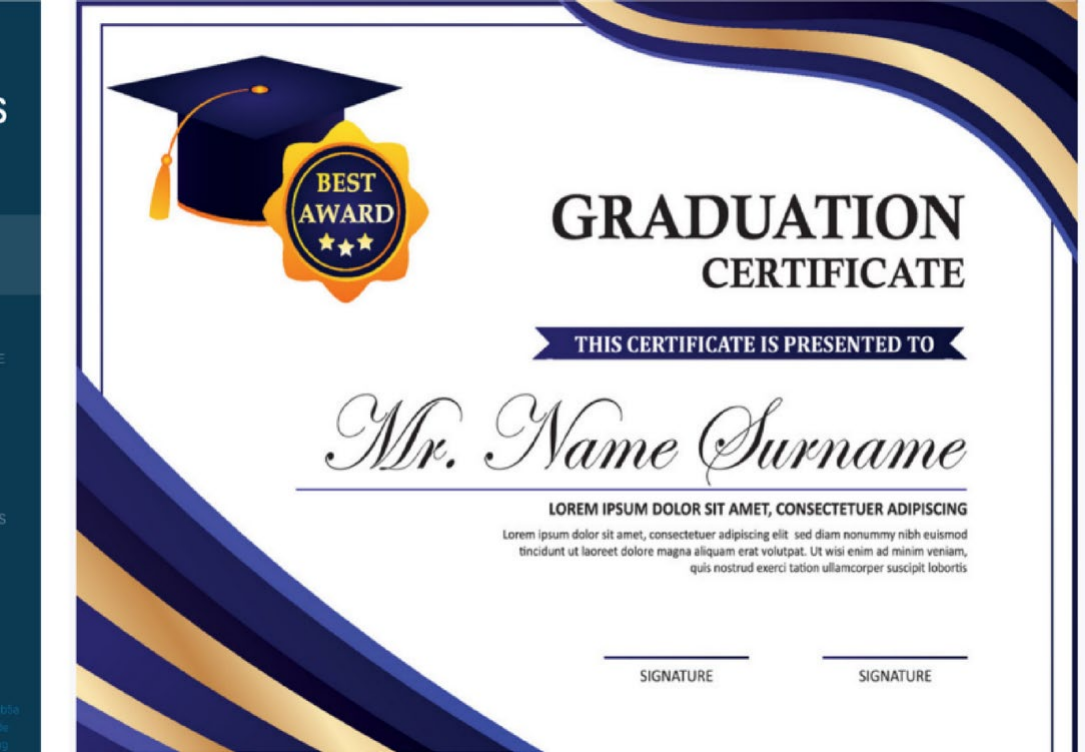
Sample of learner’s certificate.

The certificate appeared as the following Fig. 15 after its hash from IPFS.

The guest/employers can use the same steps to view the learner’s certificate or data.

As discussed in the proposed framework, any guest or employer can access the blockchain network upon approval of the university node to view the learner’s certificate and performance.

#### Test case 5: How can the learner interact with the learning activities through the smart contract?

The steps involved for the learner’s node to interact with the learning activity smart contract functions are as follows.

First, the learner can interact with the contract by inserting the contract address and the ABI of the contract to view the learning activity data (assignment file). Then, the assignment file will be viewed on the IPFS localhost after entering the hash of the file.

#### Test case 6: How can the professor view the learner’s answers on the blockchain?

After the learner’s submission of the learning activity, the transaction is broadcasted; the professor can view the submitted answers through the smart contract and its function (view answer hash). The hash of each answer file that can be viewed on the IPFS.

#### Test case 7: check the transaction validation

**Fig. 16:**
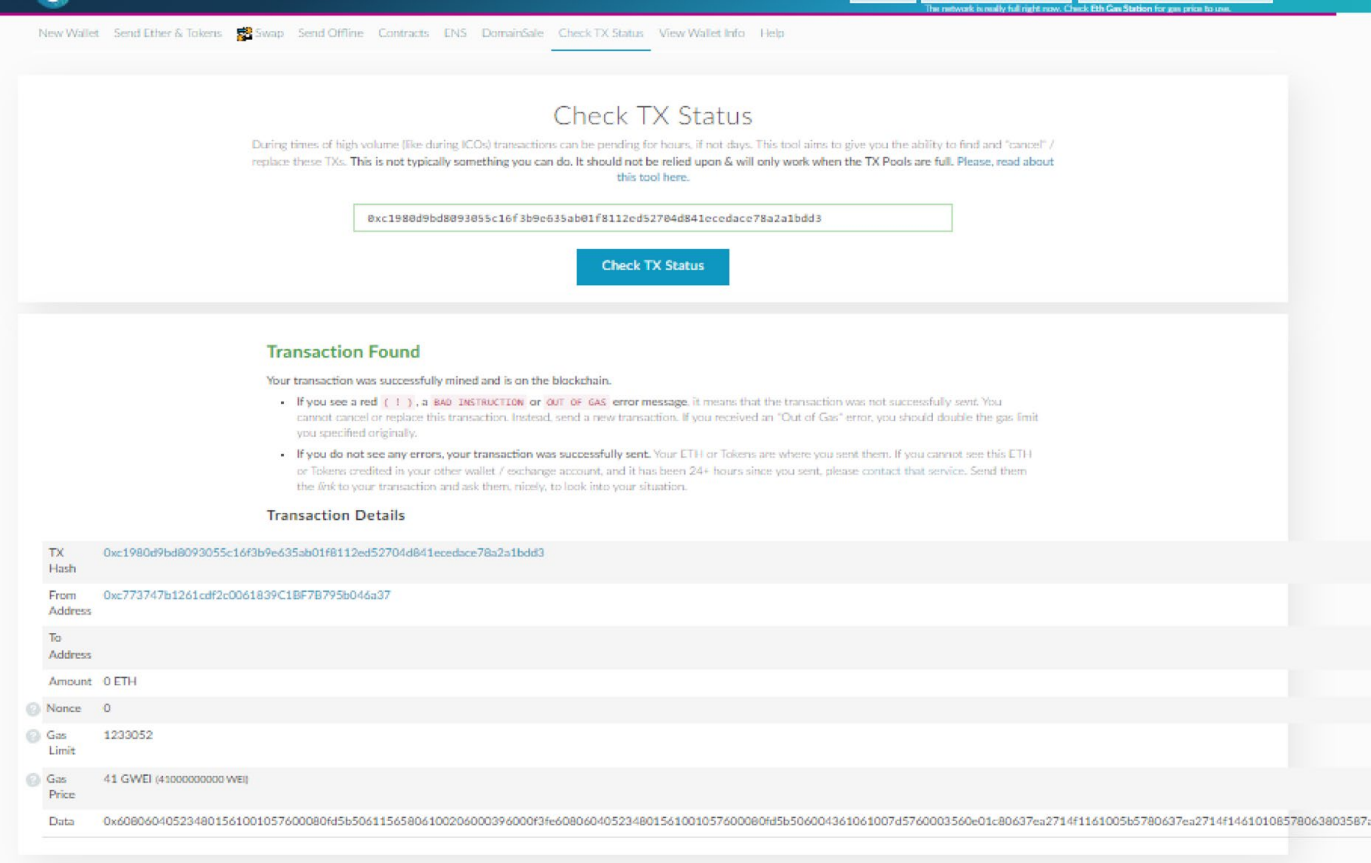
The transaction status.

The following Fig. 16 shows a sample of checking the status of the added transaction (for example, the issuance of the certificate) from the university node side on the myetherwallet platform.

#### Test case 8: check the files’ integrity in the blockchain network

**Fig. 17:**
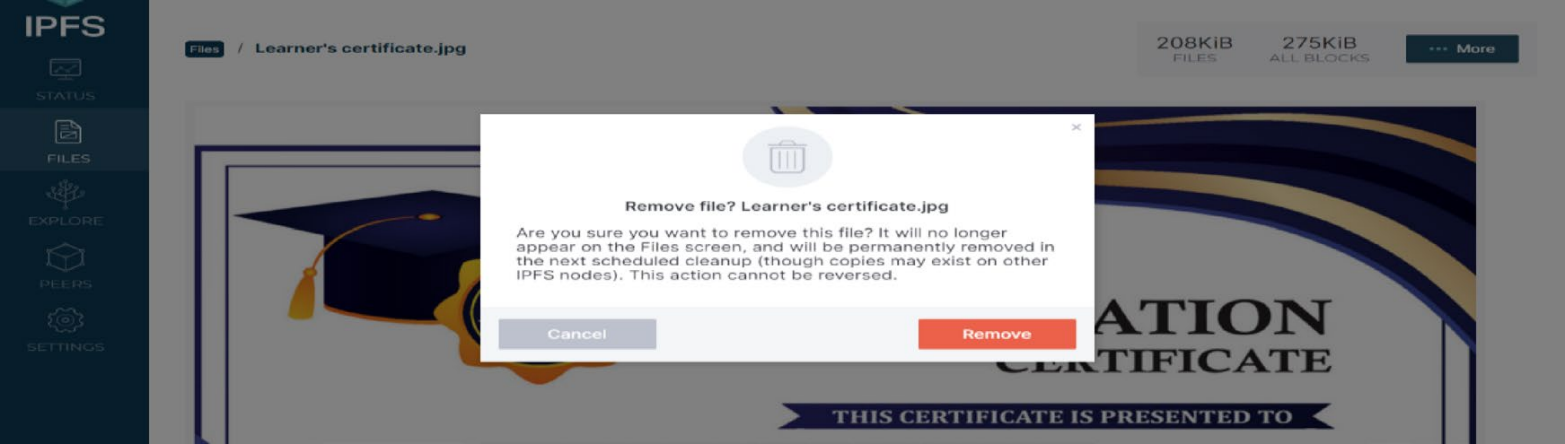
Check the file deletion.

Once the file (or any data) is uploaded into the blockchain network; because it is in a decentralized network and has appeared to all nodes in the network, it cannot be tampered with or even deleted. The following Fig. 17 checks to delete the file from the distributed network. The file will not be removed from the other nodes in the network.

To check also the integrity of the file’s content by trying to change any of the content data inside it. It is found that the hash of the file has been changed, so the file will not be accepted to be uploaded into the network.

##### Check the interaction with the smart contract functions

The following Table 13 summarizes all the contract functions and the interacting nodes, as well as showing the verification of the contract functions that were done through the Etherscan that was plugged in the Solidity remix IDE.

**Table 13:** Check smart contract functions.

Implementation scenario	Smart contract functions	Contract deployment nodes	Contract interaction nodes	Transaction status	Block added	Verification status
Upload learner’s data in the blockchain	Send hash	Univ_admin	Learner Professor Guest	Found	Yes	Verified
	Get hash					
Issue learner’s academic certificate	Issue certificate	Univ_admin		Found	Yes	Verified
	Certificate	Univ_admin	Learner Professor Guest	Found	Yes	Verified
Learning activities between the professor and the learner	Assignment	Professor	Learner	Found	Yes	Verified

#### Test case 9 and test case 10: evaluating the deep neural network model accuracy and loss

This study evaluates the proposed deep artificial neural network that uses the dense model to check model validation. Many experiments were done to get the best accuracy. First, the output is categorized into 2 categories and perform the model by utilizing the rectified linear function as the activation function and the sigmoid function for the output on this basis, but after many tries, It’s found that it is better to make the output return all 4 values at once by using the SoftMax function for the output function as well as the sparse categorical cross-entropy. Furthermore, we discovered that the more neurons and epochs there are, the higher the accuracy and, as a result, the lower the loss. The following Table 14 reviews the deep artificial neural network analysis for the accuracy and loss output for the learner’s performance prediction.

**Table 14:** Comparison between the deep neural networks activation functions’ result.

Activation function	Number of outputs	Output function	Loss function	Number of neurons	Accuracy	Loss
Rectified linear unit” Relu”	2	Sigmoid	Binary cross entropy	100	75.6%	0.60
Rectified linear unit” Relu”	2	Sigmoid	Mean squared error	100	79.8	0.25
Rectified linear unit” Relu”	4	Softmax	Sparse categorical crossentropy	300	80.1%	0.29
Rectified linear unit” Relu”	4	Softmax	Sparse categorical crossentropy	500	91.2%	0.18

**Fig. 18:**
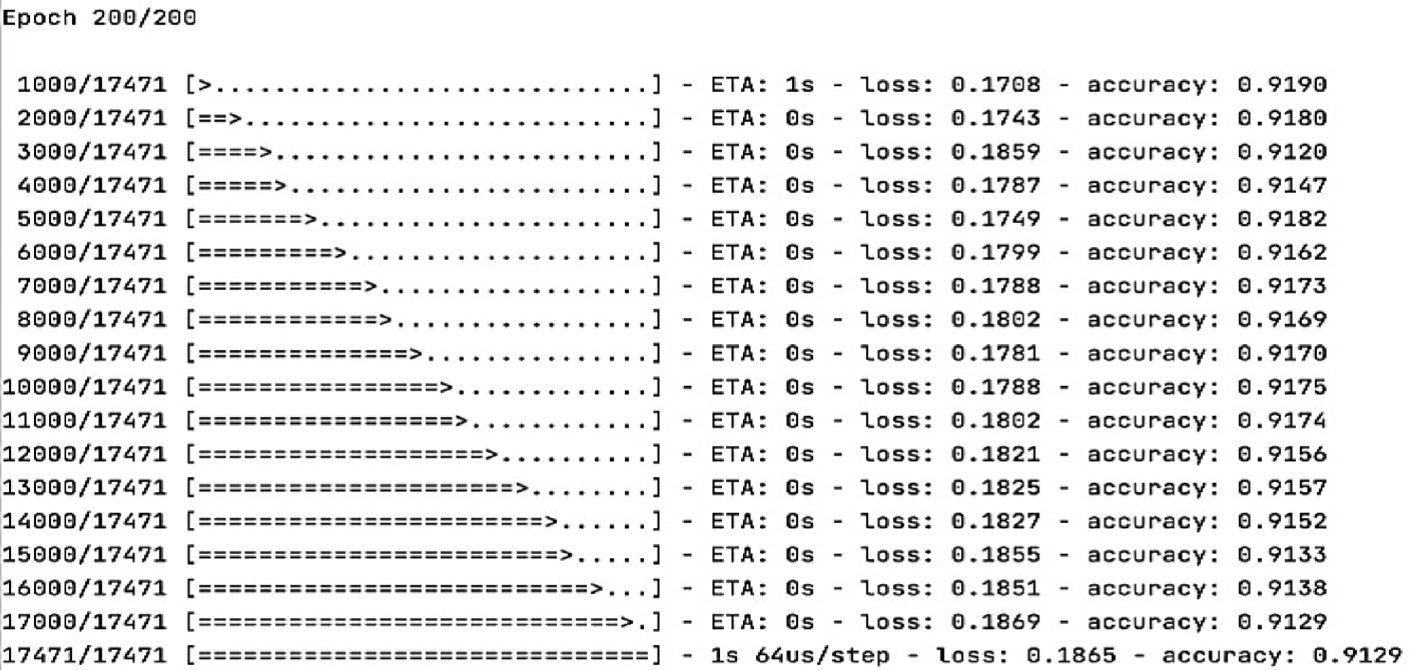
The accuracy of the proposed DNN model.

**Fig. 19:**
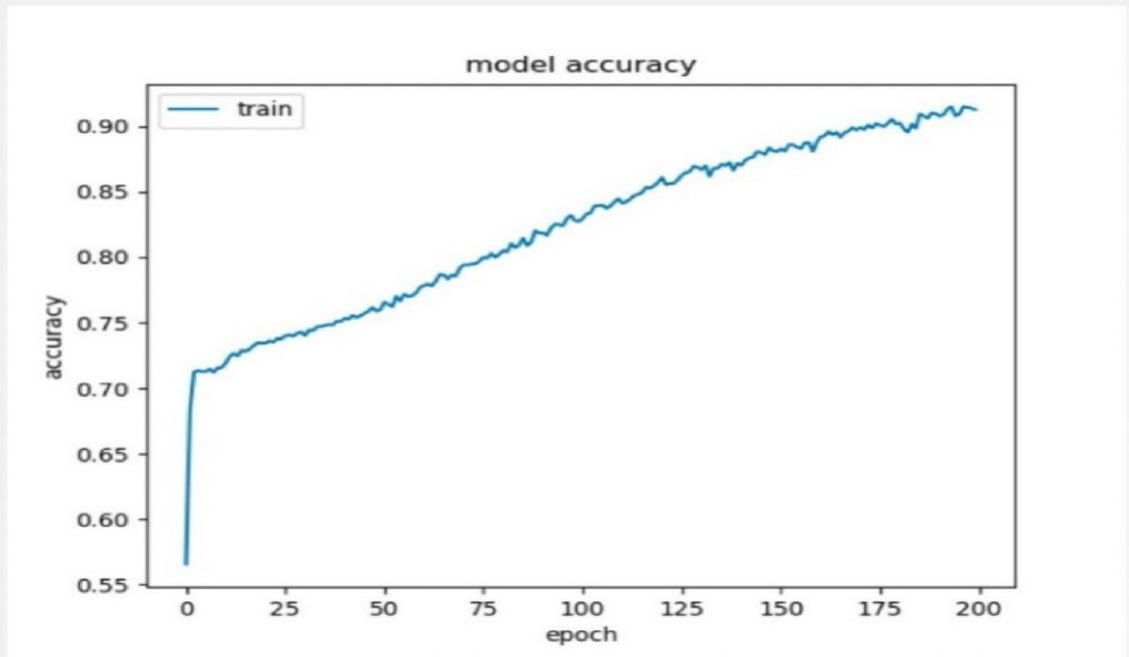
Model’s accuracy.

**Fig. 20:**
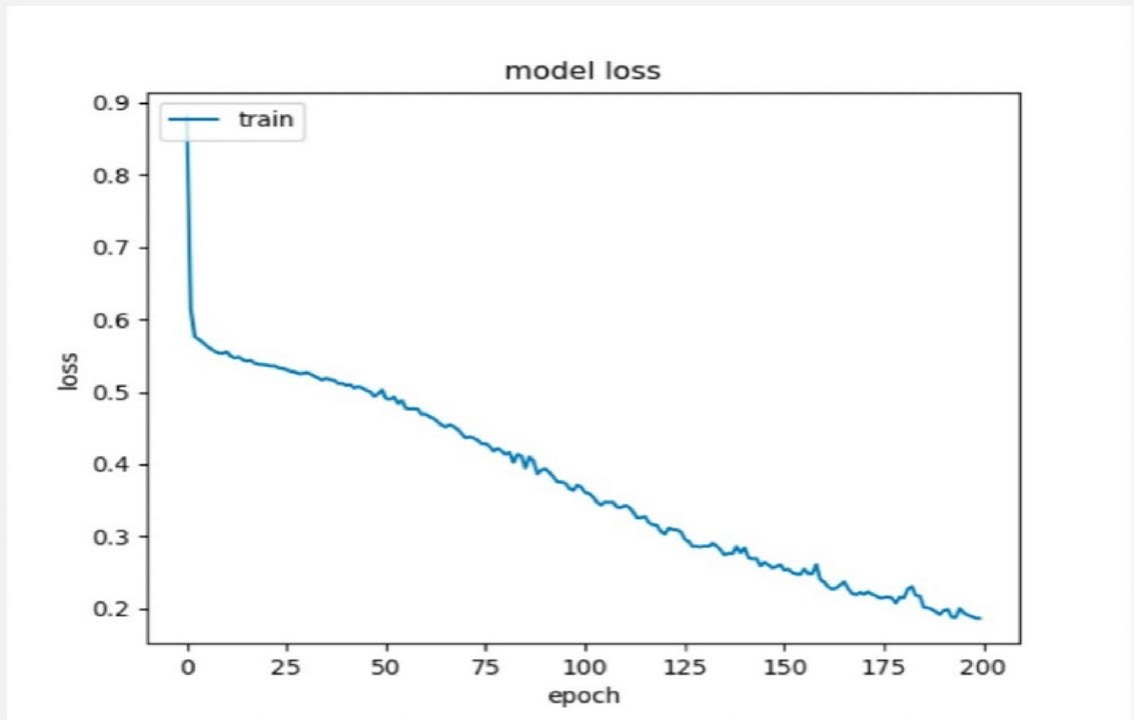
Model’s loss.

The deep artificial neural network used to predict the learner’s performance in the virtual learning environment, whether the learner status is pass, fail, make a distinction, or make an early withdrawal. The model’s accuracy is evaluated in the deep artificial neural network using the SoftMax activation function and 500 neurons for predicting the four outputs as one category. The results show high accuracy of about 91.29% and 0.18 for the loss that utilized the function of the sparse categorical cross-entropy as presented in Figs. 18, 19, and 20.

The results of the proposed model’s accuracy were considered high when compared to a study that used the same dataset, which is the open university learning analytics dataset (“OULA”), and it found that our study performed better than the other studies with the same criteria^52^.

Table 15 reviewed and compared the differences between the proposed study and the other one. It is noticed that the proposed deep neural network framework performed with high accuracy in comparison with the other study, which divided the output values into four categories and achieved an average of 86.53% for all the predictions, which is less than our proposed study. In addition, the proposed model dealt with them as one category and achieved high accuracy.

**Table 15:** The proposed study versus other study.

Study	No. of hidden layers	Output function	No. of output values	Output values	Accuracy	Loss
Waheed et al.^52^	3	Sigmoid	2	Pass/fail	84.48%	0.38
				Withdraw/pass	94.70	0.13
				Distinction/pass	80.54%	0.49
				Distinction/fail	86.40%	0.29
The proposed study	5	Softmax	4	Pass/fail/withdraw/distinction	91.29%	0.18

### Phase 4: report

In this phase, all the test cases that were presented in phase 2 and tested in phase 3 are checked to see whether they pass or fail to convert the expected result into an actual result. The following Table 16 summarizes each test case and gives a comment about the result that it achieved the desired outcome.

**Table 16:** Test cases summarize.

Test case no.	Status [pass or fail]	Comment
TC1	Pass	Nodes can interact with contract functions
TC2	Pass	Nodes can view learner’s data
TC3	Pass	Issues learner’s certificate
TC4	Pass	View learner’s certificate
TC5	Pass	Learner can view assignment file
TC6	Pass	Professor can view assignment Answer
TC7	Pass	Transaction found
TC8	Pass	Ensure the files integrity
TC9	Pass	Achieved high accuracy about 91.29%
TC10	Pass	Reached low loss about 0.18

## Result and discussion

The proposed framework’s implementation is evaluated through a set of test cases applied to check the functionality of the blockchain and the authentication of the participating nodes inside the network. After that, the transaction performance inside the block is checked to ensure whether it is found or not. The transaction status has been discovered to be “in the block”. Also, smart contract test cases were checked to verify the transactions and interactions between the nodes as well as the blockchain network. Besides, the stored data inside the blockchain is checked to ensure its integrity and it’s found that the stored files can’t be alerted or tampered and if it is deleted it will be noticed and known from its hash that will be changed from the original one. In addition, an evaluation of the deep learning model is checked, and the study indicated that all the features needed to be considered to predict the learner’s academic performance. As well as training the model, the number of hidden layers and the number of neurons play a significant role in enhancing the accuracy of the model. The study discovered that the deep neural network model predicted the learner’s performance with high accuracy as well extracting the 4 output features at the same time in contrast to other studies which played to extract the 2 output features only with an average accuracy less than our proposed study besides the secure storage of the data.

In addition, all model training and evaluation were conducted on a local Apple MacBook Air equipped with an M1 processor. The complete training process required approximately 2 h. As the computations were performed on a personal device, no additional costs associated with external computational resources were incurred.

### Research contribution

The key contribution of this research is proposing a framework for a smart learning environment based on integrating two impressive technologies like blockchain and deep learning, which is considered a novel integration, especially in the educational sector. This integration could ensure data security and transparency and provide an automated and trusted smart e-learning process.

The research focused on storing the learner’s data in the blockchain through the interplanetary file system and getting its benefits from securing the learner’s data, as well as ensuring its confidentiality and integrity. Then apply the deep learning model to this secured data to predict the learner’s performance. As well as enabling the university to issue learners’ certificates and store them in the blockchain to be available and verifiable by all the nodes in the network. Although focusing on applying the smart contract to the learning activities increases the automation of the learning process and ensures security and transparency between the learners and the professor in tasks such as assignment submission, With the blockchain and deep learning technology, the guests (employers) can trust every single piece of data recorded in the chain, as all the recorded data is immutable and the learner’s performance prediction is highly accurate. The study also discussed that integrating the blockchain with deep learning is a major challenge concerning security, where attacks can target many layers of the integration system, and each demands a unique response. Here’s an example of potential assaults and remedies at each layer that are covered in the proposed framework.

Attacks related to data layer appeared like Data poisoning which refers to introducing harmful or misleading data into a training dataset. Eavesdropping involves unauthorized access to sensitive material during transmission. These attacks were solved by Storing the training data hashes on the blockchain for immutability and integrity.

Encrypt data for safe transfer. Blockchain’s peer-to-peer network decreases the likelihood of single-point failures, making it potentially useful against DDOS attacks. Smart Contracts automate and safeguard interactions between deep learning components and the blockchain. As well as the blockchain transparency that keeps a tamperproof log of all transactions and model changes.

## Conclusion and future work

Artificial intelligence (AI) has revolutionized many services, with machine learning (ML) and deep learning (DL) as a subset. The comprehensive overview of ML, DL, and distributed systems used in many sectors for addressing key issues such as securing, data utilization, strategy, and sharing, highlighting the critical role of many sectors in community^18^. This paper discussed how to overcome some of the e-learning challenges by integrating blockchain and deep learning techniques. It discussed the implementation and creation of the university’s private Ethereum blockchain network, as well as focusing on creating nodes for all the participating users in the network and enabling them to interact through their wallets with each other through their public and private keys. Although the learners’ data was stored on the blockchain to ensure its security and validity, the study guaranteed the security of the deep learning model’s data that was distributed all over the university network. Consequently, the deep learning performance prediction model that is based on this validated, transparent data ensures the transparency of the transactions between the nodes in the university blockchain network. Also, the study ensures the integrity of the learner’s certificate that is issued by the university administration and signed by its keys. It also allowed employers to view the learner’s academic achievements and select them as appropriate candidates. The future work of the study tends to focus on enabling the learner’s tuition fees through the blockchain. Conduct online exams based on the blockchain network and auto-mark them by smart contract and provide tokens for the clever learners’ achievements as a reward. A further research point in this field of study could be related to a personalized and secure educational plan for all the educational stages of the learner, where it depends on the learner’s performance, learning style and behavior. Deep learning could help to infer this large amount of data and then record this scalable data as a learning history to be secured with the blockchain and improve the overall performance.

## Data Availability

Open University Learning Analytics Dataset (OULAD) is available on https://www.kaggle.com/datasets/anlgrbz/student-demographics-online-education-dataoulad.

## Abbreviations

${E}_{Pri}$: Employer’s private key
${E}_{Pub}$: Employer’s public key
${L}_{Pri}$: Learner’s private key
${L}_{Pub}$: Learner’s public key
${P}_{Pri}$: Professor’s private key
${P}_{Pub}$: Professor’s public key
${U}_{Pri}$: University private key
${U}_{Pub}$: University public key
ABI: Application binary interface
AI: Artificial intelligence
ANN: Artificial neural network
BTC: Bitcoin
CNN: Convolutional neural networks
DBN: Deep belief network
DL: Deep learning
UNESCO: United Nations Educational, Scientific, and Cultural Organization
VLE: Virtual learning environment
DNN: Deep neural network
ETH: Ethereum
EVM: Ethereum virtual machine
GPA: Grade point average
IDE: Integrated development environment
IOT: Internet of Things
IPFS: Interplanetry file system
JSON: JavaScript object notation
MEW: MyEtherWallet
ML: Machine learning
MLP: Multilayer perceptron
MOOC: Massive open online courses
OULA: Open university learning analytics
PCA: Principal component analysis
Relu: Rectified liner unit
